# The balancing role of distribution speed against varying efficacy levels of COVID-19 vaccines under variants

**DOI:** 10.1038/s41598-022-11060-8

**Published:** 2022-05-06

**Authors:** Daniel Kim, Pınar Keskinocak, Pelin Pekgün, İnci Yildirim

**Affiliations:** 1 H. Milton Stewart School of Industrial and Systems Engineering, Georgia Institute of Technology, North Ave NW, Atlanta, GA 30332 USA; 2 Moore School of Business, University of South Carolina, Columbia, SC 29208 USA; 3 Department of Pediatrics, Section of Infectious Diseases and Global Health, Yale School of Medicine, Yale Institute of Global Health, 1 Church Street, New Haven, CT 06510 USA

**Keywords:** Health policy, Applied mathematics

## Abstract

During a pandemic, vaccination plays an important role in reducing the infection spread or adverse outcomes such as hospitalizations and deaths. However, a vaccine’s overall public health impact depends not only on its initial efficacy, but also its efficacy against emerging variants and ease and speed of distribution. For example, mutations in SARS-CoV-2 raised concerns about diminishing vaccine effectiveness against COVID-19 caused by particular variants. Furthermore, due to supply-chain challenges, the accessibility and distribution of the vaccines have been hindered in many regions, especially in low-income countries, while the second or third wave of the COVID-19 pandemic has occurred due to the variants. Hence, we evaluated the interactions between the *speed of distribution* and *efficacy against infection* of multiple vaccines when variants emerge by utilizing a Susceptible-Infected-Recovered-Deceased model and assessing the level of *infection attack rate*. Our results show that speed is a key factor to a successful immunization strategy to control the pandemic even when the emerging variants may reduce the efficacy of a vaccine. Understanding the interactions between speed and efficacy and distributing vaccines that are available as quickly as possible are crucial to eradicate the pandemic before new variants spread.

## Introduction

Since the initial reports of a cluster of pneumonia cases of unidentified origin in Wuhan, China, in December 2019, more than 483 million people around the world have been infected with the novel severe acute respiratory syndrome coronavirus 2 (SARS-CoV-2)^1^. Despite the development of effective vaccines in unprecedented speed, concerns have been raised on the potential reduction in efficacy of these vaccines against the new SARS-CoV-2 variants due to possible evasion from antibody recognition^2,3^. While governments and policymakers may favor a high-efficacy vaccine during a pandemic, the overall public health impact of vaccination also depends on whether a vaccine can be dispensed quickly and/or the change in its efficacy against the emerging variants compared to other vaccines. Hence, the goal of this study was to understand the interactions between the speed of distribution vs. the change in the efficacy levels of vaccines against infection before and after the emergence of variants, which we refer to as “initial efficacy” and “final efficacy,” respectively.

The procurement and dissemination of the SARS-CoV-2 vaccines and ancillary supplies (e.g., glass vials used for storage^4^) have posed significant global challenges, particularly for low-income countries^5^. In addition, the high-efficacy mRNA vaccines for COVID-19 require ultra-cold storage and logistics, which are often not readily available or easy to acquire, especially in low-income countries^6,7^. Due to such challenges, as of March 2022, only 14.5% of people in low-income countries (64.4% of the world population) have received at least one dose of the vaccines^8,9^.

The distributional challenges and delays lead to continuous infections, providing an opportunity to the variants of the virus to emerge, which has raised concerns regarding reduced efficacy of vaccines against emerging variants^10^. As of March 2022, five concerning SARS-CoV-2 variants have been identified: B.1.1.7, B.1.351, P.1, B.1.617.2, and B.1.1.529^11^. These have been classified as the variants of concern (VOC) because they have quickly become dominant^12–15^. The variant B.1.1.529 (a.k.a. omicron), for instance, was first detected in the U.S. on December 1st, 2021 and classified as a dominant variant on December 20th, 2021^16^. These variants became more alarming as multiple studies showed that the effectiveness of various vaccines decreased against the variants^17–20^.

In this paper, we studied the interactions between vaccines’ efficacy levels, which often reduce due to emerging variants, and the speed of vaccine distribution, during a pandemic. We developed a Susceptible-Infected-Recovered-Deceased (SIR-D) simulation model and assessed the *infection attack rate* (IAR, i.e., the percentage of the population that contracts the disease during a certain time horizon) under various variant emergence times. Prior studies used modified SIR-D models to study the impact of public health interventions, including social distancing and vaccination^21–25^. While some studies examined the interactions between vaccine efficacy and distribution speed^24,25^, to the best of our knowledge, this is the first study to consider the impact of a change in vaccine efficacy due to the emergence of variants during a pandemic. Throughout this paper, to the term vaccine distribution refers to the entire distribution process of a vaccine including delivery to the dispensation sites and administration to the population. The results of this study are aimed to guide decision-makers in vaccine ordering during a pandemic when there are multiple types of vaccines, facing reduced efficacies as variants emerge.

## Methods

We developed an SIR-D model (referred to as the main model), conducted extensive sensitivity analysis on the main model, and also developed an extended SIR-D model (described in Supplemental Materials) to capture additional details of the disease dynamics.

### Vaccine efficacy and capacity

To compare different vaccine types, we categorized the level of the vaccine efficacy into three ranges: “H” (High) if 90% or above, M (Moderate) if 70% or above and lower than 90%, and L (Low) if lower than 70%. We assumed that the final efficacy was always lower than the initial efficacy. We considered three initial efficacy levels ($H_{i} =$ 95%, $M_{i} =$ 75%, and $L_{i} =$ 65%) and three final efficacy levels ($H_{f} =$ 90%, $M_{f} =$ 70%, and $L_{f} =$ 60%). Consequently, we obtained six types of vaccines, defined by a particular initial and final efficacy, as summarized in Table 1. These modeling choices were motivated by recent studies on vaccine efficacy against variants^26–28^.

**Table 1:** Vaccine efficacy.

Vaccine type	Initial efficacy (%)	Final efficacy (%)
$H_{i} H_{f}$	95	90
$H_{i} M_{f}$	95	70
$H_{i} L_{f}$	95	60
$M_{i} M_{f}$	75	70
$M_{i} L_{f}$	75	60
$L_{i} L_{f}$	65	60

In the simulations, a single type of vaccine was administered, and all vaccines required a single dose. In each simulation, the daily vaccine distribution capacity was kept constant at $\lambda \cdot K$, where $K$ represents base capacity and $\lambda$ is a multiplier. We fixed the base capacity, $K,$ at 500,000, motivated from the average number of vaccine recipients in each day in the United States from December 14, 2020 to March 2, 2021, and we set a range of 1.0 to 3.0 with increments of 0.2 for the capacity multiplier $\lambda$ to represent the speed of distribution in the simulations^29^.

### Main SIR-D model

**Figure 1:**
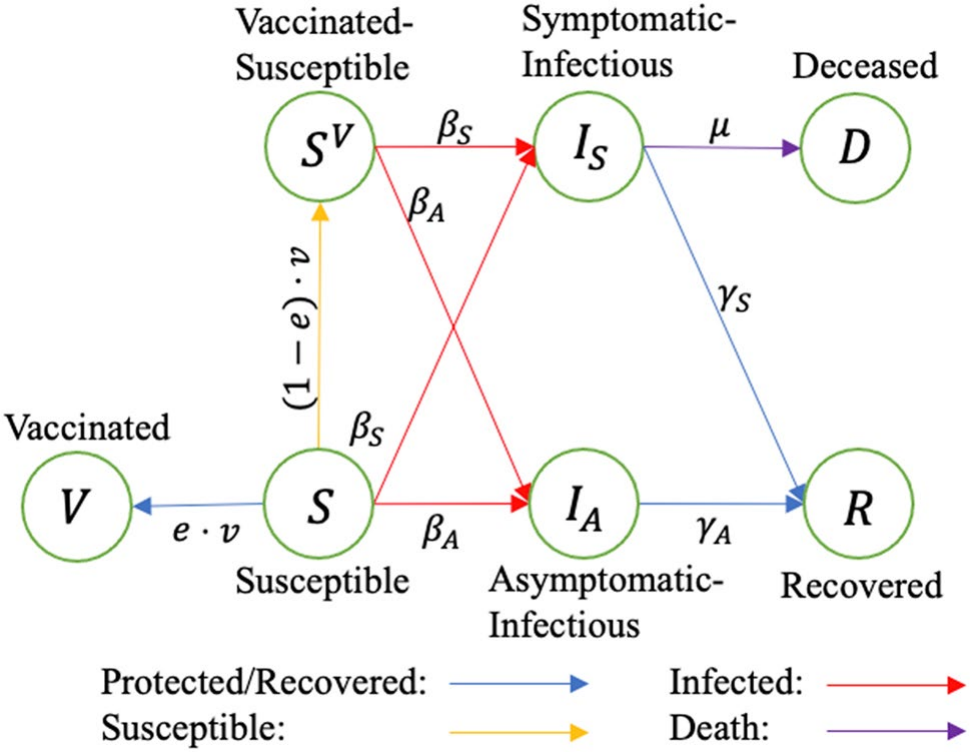
Transition diagram of the main SIR-D model, in which each move is dependent on various parameters. $\beta_{S} ,\;\beta_{A}$: symptomatic and asymptomatic transmission rates, respectively; $\gamma_{S} ,\;\gamma_{A}$: symptomatic and asymptomatic recovery rates, respectively; μ: decease rate of a symptomatic patient; $e$: efficacy of the vaccine; $v$: daily vaccinated.

In this study, we adapted an SIR-D (Susceptible-Infectious-Recovered-Deceased) compartmental model, where individuals move among compartments, and transitions between compartments are governed by ordinary differential equations given epidemiological and vaccine parameters. We implemented seven compartments: *Susceptible* ($S$), *Vaccinated with immunity* ($V$), *Vaccinated-susceptible* ($S^{V}$), *Symptomatic-infected* ($I_{S}$), *Asymptomatic-infected* ($I_{A}$), *Recovered* ($R$), and *Deceased* ($D$). When *Susceptible* population received vaccines, they entered either the *Vaccinated with immunity* ($V$) compartment if the vaccine was effective, or the *Vaccinated-susceptible* ($S^{V}$) compartment, otherwise. The *Vaccinated with immunity* ($V$) population became fully protected against the disease upon vaccination (i.e., time-to-immunity was zero). We also conducted sensitivity analyses on the main model by varying the time-to-immunity from several days to weeks. Both Susceptible and Vaccinated-susceptible populations transitioned to either the *Symptomatic-infected* ($I_{S}$) or *Asymptomatic-infected* ($I_{A}$) compartment, after becoming infected. Symptomatic-infectious population then moved to either the *Recovered* ($R$) or *Deceased* ($D$) compartment. We assumed that asymptomatic patients always recovered. The transition diagram of the main SIR-D model is depicted in Fig. 1.

We chose the epidemiological parameter based on the estimated SARS-CoV-2 characteristics in various studies. The infectious periods of symptomatic and asymptomatic patients were 14 days and 8 days, respectively^30,31^. Therefore, we set the recovery rates of symptomatic and asymptomatic patients at $\gamma_{S} =$ 1/14 and $\gamma_{A} =$ 1/8, respectively. In addition, following the CDC COVID-19 Pandemic Planning Scenarios^32^, we used 2.5 for the reproduction number ($R_{0}$) and assumed that 70% of the infected individuals became symptomatic. We set the death rate $\mu$ = 0.0015 and the symptomatic-transmission rate $\beta_{S}$ = 0.219328, given the reproduction number. We assumed that the asymptomatic-transmission rate $\beta_{A}$ = 75% of the symptomatic-transmission rate^33^. We used R-software to run the simulations with a population size ($N$) of 330 million (approximate population of the United States). Since our main goal was to analyze the interactions between distribution speed and vaccine efficacy under variants, we started the simulation only after when the vaccine became available and initialized it such that around 5.036% of the population had already been infected. Thus, we set 0.504% of the population as symptomatic-infected, 0.192% as asymptomatic-infected, 4.248% as recovered, and 0.092% as deceased. These estimates were motivated by the confirmed cumulative cases and deaths as of December 14, 2020, the first day of vaccine distribution in the United States^34^. The initial values in other compartments were estimated using the epidemiological parameters defined previously. All parameters used in the main SIR-D model are summarized in Table 2 and the non-linear system of ordinary differential equations (ODEs) is as follows:

$$\begin{aligned} \frac{dS}{{dt}} & = - \left( {v + \beta_{S} \left( {1 - {\text{v}}} \right)I_{S} + \beta_{A} \left( {1 - {\text{v}}} \right)I_{A} } \right)S \\ \frac{dV}{{dt}} & = e \cdot v \cdot S \\ \frac{{dS^{V} }}{dt} & = \left( {1 - e} \right) \cdot v \cdot S - \left( {\beta_{S} I_{S} + \beta_{A} I_{A} } \right) \cdot S^{V} \\ \frac{{dI_{S} }}{dt} & = p_{S} \cdot \left( {\beta_{S} \left( {1 - v} \right)I_{S} + \beta_{A} \left( {1 - v} \right)I_{A} } \right) \cdot S + p_{S} \cdot \left( {\beta_{S} I_{S} + \beta_{A} I_{A} } \right) \cdot S^{V} - \gamma_{S} I_{S} - \mu_{S} I_{S} \\ \frac{{dI_{A} }}{dt} & = (1 - p_{S} ) \cdot \left( {\beta_{S} \left( {1 - v} \right)I_{S} + \beta_{A} \left( {1 - v} \right)I_{A} } \right) \cdot S + (1 - p_{S} ) \cdot \left( {\beta_{S} I_{S} + \beta_{A} I_{A} } \right) \cdot S^{V} - \gamma_{A} I_{A} \\ \frac{dR}{{dt}} & = \gamma_{S} I_{S} + \gamma_{A} I_{A} \\ \frac{dD}{{dt}} & = \mu_{S} I_{S} \\ \end{aligned}$$

**Table 2:** Parameters used in the main SIR-D model.

Parameter	Description	Value
$\beta_{S} ,\beta_{A}$	Transmission rate	$0.219328,\; 0.164496$
$\gamma_{S} ,\gamma_{A}$	Recovery rate	$1/14, 1/8$
$\mu$	Death rate	$0.0015$
$N$	Population size	330 million
$p_{S}$	Probability of developing symptoms	0.7
$e$	Vaccine efficacy	See Table 1
$K$	Base capacity	500,000
$\lambda$	Capacity multiplier	1.0 to 3.0 with increments of 0.2
$v$	Daily vaccinated individuals	${\text{min}}\left( {1,\frac{\lambda \cdot K}{{S \cdot N}}} \right)$

We ran the simulation on a one-year time horizon under different *mutation times* (i.e., the time at which the emerging variant reduces a vaccine’s efficacy) within the range of day 5 to day 60 with a discrete step size of 5 days, and different *capacity multipliers* ($\lambda$) within the range of 1.0 to 3.0 with a discrete step size of 0.2 to capture the vaccine distribution speed.

### Extended SIR-D model

We extended the main SIR-D model described above where infected populations were differentiated by their vaccination status and some of the vaccinated individuals became susceptible again after the mutation time. These extensions also reflected the reduction in mortality risk observed in vaccinated populations compared to unvaccinated populations. The details of the extended SIR-D model are presented in Supplementary Materials.

## Results

We first ran the simulations in the absence of vaccines, resulting in an estimated IAR of approximately 88.93% and a mortality rate (i.e., the percentage of the population that dies from the disease during the time horizon) of 1.3%. The daily infection peak (i.e., the highest percentage of the population who get newly infected on a single day) occurred on day 41, at which 0.624% of the population got newly infected.

**Figure 2:**
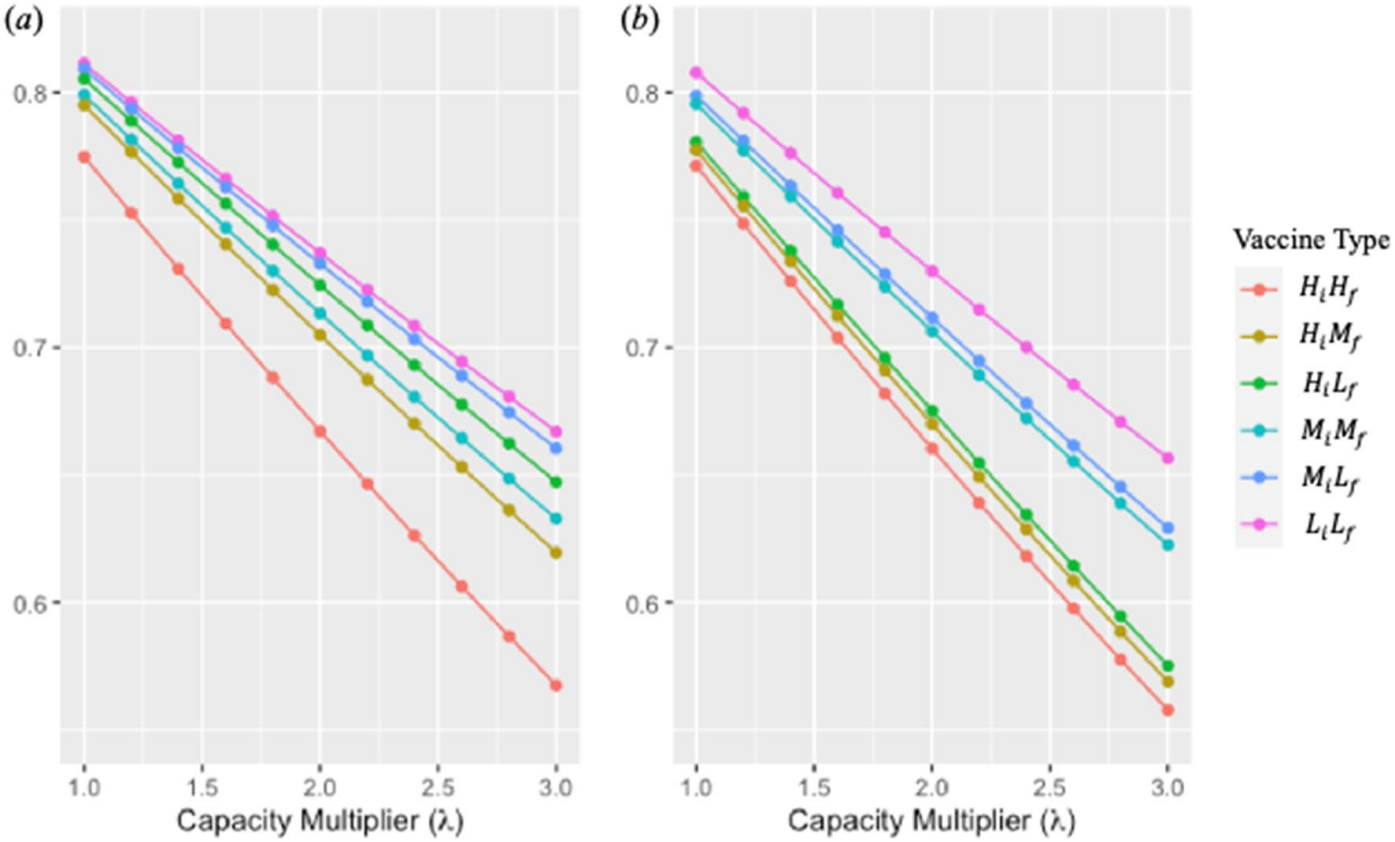
Infection attack rate under different capacity multipliers with different vaccine types when mutation time is (**a**) day 10 and (**b**) day 50.

Table 3 and Fig. 2 show the estimated IAR under different capacity multipliers ($\lambda$) when the mutation times are day 10 and day 50. We report the IAR and mortality rate with different mutation times in Supplementary Materials (Tables S1 and S2). We observe that IAR decreases as the mutation time occurs later and/or the capacity multiplier increases. When increasing the capacity multiplier, the reduction in IAR is larger when the mutation time is early (e.g., day 10) versus late (e.g., day 50). In addition, even vaccine-$L_{i} L_{f}$ can achieve a lower IAR than vaccine-$H_{i} H_{f}$ if the capacity multiplier of vaccine-$L_{i} L_{f}$ is high compared to that of vaccine-$H_{i} H_{f}$. We present the minimum required capacity multiplier of all vaccine types to achieve a lower IAR than vaccine-$H_{i} H_{f}$ under different mutation times in Supplementary Materials.

**Table 3:** Infection attack rate (%) under different capacity multipliers and vaccine types when mutation time is day 10 and day 50.

Capacity multiplier ($\lambda$)	Mutation time = Day 10						Mutation time = Day 50					
	$H_{i} H_{f}$	$H_{i} M_{f}$	$H_{i} L_{f}$	$M_{i} M_{f}$	$M_{i} L_{f}$	$L_{i} L_{f}$	$H_{i} H_{f}$	$H_{i} M_{f}$	$H_{i} L_{f}$	$M_{i} M_{f}$	$M_{i} L_{f}$	$L_{i} L_{f}$
3	56.75	61.95	64.72	63.28	66.04	66.70	55.77	56.89	57.51	62.25	62.91	65.65
2.8	58.67	63.61	66.23	64.85	67.45	68.06	57.75	58.85	59.46	63.88	64.52	67.08
2.6	60.63	65.30	67.76	66.43	68.88	69.44	59.76	60.84	61.43	65.54	66.15	68.53
2.4	62.62	67.01	69.30	68.05	70.33	70.84	61.81	62.86	63.44	67.21	67.80	70.00
2.2	64.65	68.73	70.86	69.68	71.79	72.26	63.90	64.91	65.46	68.91	69.47	71.49
2	66.71	70.48	72.43	71.33	73.28	73.70	66.03	66.99	67.51	70.63	71.16	72.99
1.8	68.81	72.25	74.02	73.00	74.77	75.15	68.18	69.09	69.58	72.37	72.87	74.51
1.6	70.93	74.03	75.63	74.70	76.29	76.62	70.37	71.22	71.68	74.14	74.59	76.05
1.4	73.08	75.84	77.25	76.41	77.82	78.10	72.59	73.37	73.78	75.92	76.33	77.61
1.2	75.27	77.66	78.88	78.15	79.36	79.61	74.85	75.54	75.91	77.72	78.09	79.18
1	77.48	79.50	80.53	79.90	80.93	81.13	77.13	77.73	78.05	79.55	79.87	80.77

**Figure 3:**
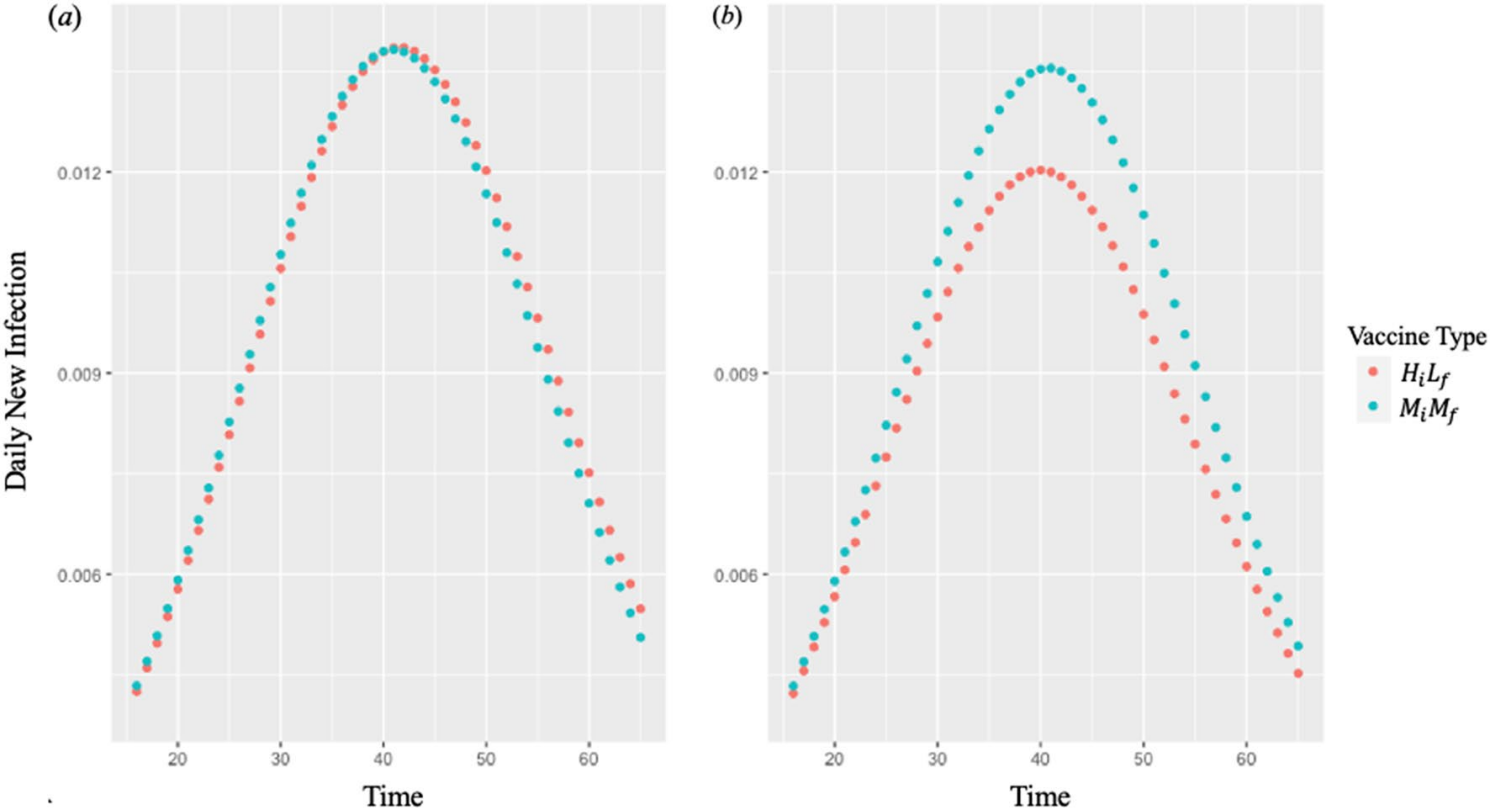
Daily new infections from day 15 to day 65 with vaccine-$H_{i} L_{f}$ and vaccine-$M_{i} M_{f}$ when the capacity multiplier ($\lambda$) is 3 and the mutation time is (**a**) day 10 and (**b**) day 50.

When the mutation time is early and before the daily infection peak, vaccine-$M_{i} M_{f}$ achieves a lower IAR than vaccine-$H_{i} L_{f}$ for all capacity multipliers (Table 3). Figure 3 compares the daily new infections from day 15 to day 65 with vaccine-$H_{i} L_{f}$ vs. vaccine-$M_{i} M_{f}$ when the capacity multiplier is 3 and the mutation time is day 10 and day 50. After the daily infection peak is reached, the number of daily infections drops at a faster rate when vaccine-$M_{i} M_{f}$ is administered and the mutation time is day 10. On the other hand, when the mutation time is after the daily infection peak (e.g., day 50), the administration of vaccine-$H_{i} L_{f}$ results in a lower daily infection peak than that of vaccine-$M_{i} M_{f}$ throughout the time horizon. We provide the daily new infections for all vaccine types in Supplementary Materials.

The simulation results of the sensitivity analysis of the time-to-immunity in the main model and the extended SIR-D model are reported in Supplementary Materials. For both settings, we observe results with a similar pattern as in the main model. Compared to the main scenario, both the IAR and mortality rate increase as the time-to-immunity increases. In the extended model, a delay in the mutation time increases the IAR if the mutation occurs before the daily infection peak and decreases the IAR if the mutation occurs after the daily infection peak. This is in contrast to the scenarios in the main model where a delay in the mutation dime always decreases the IAR.

## Discussion

In this study, we developed an SIR-D model to simulate the trajectory of an infectious disease, considering different kinds of vaccines virus mutation times, where the vaccines’ efficacies decrease against the variants. The results suggest that the speed of the vaccine distribution is a key factor to achieve low IAR levels, along with vaccine efficacy both before and after the variants emerge.

Our study showed that a vaccine with low initial and final efficacy (vaccine-$L_{i} L_{f}$) could achieve a lower IAR than a vaccine with high initial and final efficacy (vaccine-$H_{i} H_{f}$) if the former could be distributed more quickly than the latter, regardless of the mutation time. In our main model, when the capacity multiplier of vaccine-$H_{i} H_{f}$ was 1.0 (0.5 M doses/day) and the mutation time was day 50 (i.e., 9 days after the daily infection peak), 77.13% of the population got infected. If the capacity multiplier of vaccine-$L_{i} L_{f}$ was at least 1.47 (0.735 M doses/day or higher), less than 77.13% of the population was infected (Tables 3 and S5). In the extended model in which some vaccinated individuals became susceptible after the mutation, the insights remained similar; the number of infected individuals under vaccine-$L_{i} L_{f}$ with a capacity multiplier of at least 1.6 was lower than that under vaccine-$H_{i} H_{f}$ with a capacity multiplier of 1.0 (Table S7). Since the start of the COVID-19 vaccination, there were several challenges in vaccine distribution. Despite the continuing effort of increasing production capacities, vaccine manufacturers, especially those who produce mRNA vaccines, have been struggling to secure sufficient supply of vaccine ingredients, storage containers, and more, due to the demand from billions of people around the world^35^. In addition, mRNA vaccines need to be stored in ultra-cold freezers under specific expiration dates, although many communities, especially in the low-income countries, lack or cannot afford such infrastructure, leading to a limited number of administration sites. Besides the mRNA vaccines, other COVID-19 vaccines that require distribution resources similar to that of the seasonal flu vaccine have been developed and administered throughout the world. These vaccines may reduce vaccine wastage, enable efficient production and distribution using the existing vaccine supply chain, and facilitate a faster rate of vaccination^36^. Hence, despite having lower efficacy than mRNA vaccines, other vaccines may have the potential for faster distribution and positive public health impact.

Increasing the doses distributed per day, i.e., the capacity multiplier ($\lambda$), of any vaccine type reduces the IAR, with the largest impact observed for vaccine-$H_{i} H_{f}$. In particular, when the capacity multiplier increases from 1.0 to 3.0, under vaccines $H_{i} H_{f}$, $M_{i} M_{f}$, and $L_{i} L_{f}$, IAR decreased from 77.13 to 55.77%, from 79.55 to 62.25%, and from 80.77 to 65.65%, respectively, when the difference between the initial and final efficacy was 5% for all the vaccine types and the mutation time was day 50. In addition, if vaccine-$H_{i} H_{f}$ could be distributed at a faster rate, the minimum required capacity multiplier ($\lambda$) of vaccine-$L_{i} L_{f}$ to achieve a lower IAR than vaccine-$H_{i} H_{f}$ was even larger (Table S5). For example, when the mutation time was day 50, the capacity multiplier of vaccine-$L_{i} L_{f}$ needed to be at least 1.47 to achieve a lower IAR than vaccine-$H_{i} H_{f}$ with the capacity multiplier of 1.0. On the other hand, when the vaccine-$H_{i} H_{f}$’s capacity multiplier was 2.0, the capacity multiplier of vaccine-$L_{i} L_{f}$ needed to be at least 2.95. Thus, even though the difference in the capacity multiplier of vaccine-$H_{i} H_{f}$ was only 1.0, that of vaccine-$L_{i} L_{f}$ was 1.48 (i.e., $2.95 - 1.47$). However, increasing the capacity multiplier, i.e., the speed of distribution, for vaccine-$H_{i} H_{f}$, may be much more challenging than that for vaccine-$L_{i} L_{f}$, considering the economic burden and limited distribution infrastructure. Hence, in some settings, it may be more beneficial to allocate resources towards distributing a lower efficacy vaccine at a faster rate, as our study shows.

Forecasting the time when the peak infections occur and when the variants emerge is also critical to choosing which type of vaccine to implement as the main intervention for maximizing public health benefits. An effective vaccination program achieves the highest reduction in the number of new infections *before* the daily infection peak^37^. Afterwards, even with a higher efficacy, a vaccine cannot reduce the size of the susceptible population as much as before the daily infection peak. Consequently, if the mutation time comes before the daily infection peak, the vaccine with a high final efficacy, despite its lower initial efficacy, achieves a lower IAR than the vaccine with a higher initial efficacy. For example, when we compared vaccine-$H_{i} L_{f}$ and vaccine-$M_{i} M_{f}$ with a capacity multiplier of 3.0 for each, the daily infection peak occurred on day 40 and 41, respectively. Then, under the mutation time of day 10, the administration of vaccine-$M_{i} M_{f}$, which had an initial efficacy of 75% and final efficacy of 70%, resulted in an IAR of 63.28%, whereas the administration of vaccine-$H_{i} L_{f}$, which had an initial efficacy of 95% and final efficacy of 60%, resulted in an IAR of 64.72% (Table 3, Fig. 3). A delay in the mutation time decreases the IAR in the main model since more people become protected with the vaccine’s higher efficacy before the emergence of variants. In the extended model considering the waning of immunity due to variants, the insights remained similar; under the mutation time of day 10 and the capacity multiplier for 3.0, the administration of vaccine-$H_{i} L_{f}$ resulted in an IAR of 62.94% and that of vaccine-$M_{i} M_{f}$ resulted in an IAR of 62.37% (Table S7). In contrast to the main model, a delay in the mutation time increases the IAR if the mutation occurs before the daily infection peak and decreases the IAR if it occurs after the daily infection peak in the extended model. When the mutation occurs, vaccinated people become re-susceptible to infection due to variants, and their risk of infection increases with a growing number of the infected population (Fig. S3). Active genomic surveillance that studies the evolvement of the virus is critical to identify a new variant and determine its influence on the spread of the disease and the vaccine efficacy^38^. However, genomic surveillance has not received as much attention, and the coverage is still low^39,40^. Our results demonstrate that an expedited detection of the variants and their timings are vital to the choice of a vaccine to minimize the IAR.

### Limitation

We acknowledge some limitations of this study. Our compartmental model provided insights on the interactions between speed and efficacy against emerging variants without confounding the impact of other interventions. However, it can be extended to capture more realistic trajectory of SARS-CoV-2, including more compartments or time-dependent epidemiological parameters^41,42^. For instance, we assumed that every type of vaccine requires a single dose. In practice, the majority of the authorized vaccines require two doses with three to four weeks apart application and it may take several days to gain immunity after vaccination.

The study can be extended for future research to model the simultaneous deployment of a portfolio of vaccines and/or non-pharmaceutical interventions (e.g., mask mandates). Studying the resource allocations that maximize their synergies and provide better health outcomes under the presence of variants can additionally guide decision-makers in vaccine ordering.

## Conclusion

Overall, our results suggested that the administration of a vaccine with high efficacy against both the original strain and the variants may not always lead to a low number of cumulative infections if it cannot be distributed as quickly as other vaccine types with lower efficacies. Despite the vast efforts for worldwide vaccination, vaccine distribution has been an ongoing challenge due to production shortages, economic constraints, and the lack of advanced supply-chain infrastructure, which is critical in effective distribution of the high-efficacy vaccines. Due to these challenges, the accessibility and distribution of the vaccines have been hindered, even more than a year after the vaccines were developed, especially in many low- and middle-income countries^43–45^. It is critical to distribute available vaccines as quickly as possible and vaccinate more people to reach herd immunity before new variants spread. Our study demonstrated that a vaccine with a relatively lower efficacy can achieve at least as good health outcomes as their higher efficacy counterparts, as long as it can be distributed more quickly. We hope that our study provides guidance to decision makers on the interactions between speed and efficacy, highlighting the critical role of speed of vaccination during a pandemic as variants that decrease efficacy of vaccines emerge.

## Supplementary Information

Supplementary Information.
