# Supplementary material for: The balancing role of distribution speed against varying efficacy levels of COVID-19 vaccines under variants
Source: Sci Rep. 2022 May 6;12:7493. doi: 10.1038/s41598-022-11060-8 (PMC9075929; doi:10.1038/s41598-022-11060-8)
Supplement: Supplementary file 1 — Supplementary Information. [file 41598_2022_11060_MOESM1_ESM.docx]

**SUPPLEMENTARY MATERIALS**

1. **Main Model – Daily new infections with all vaccine types**

**
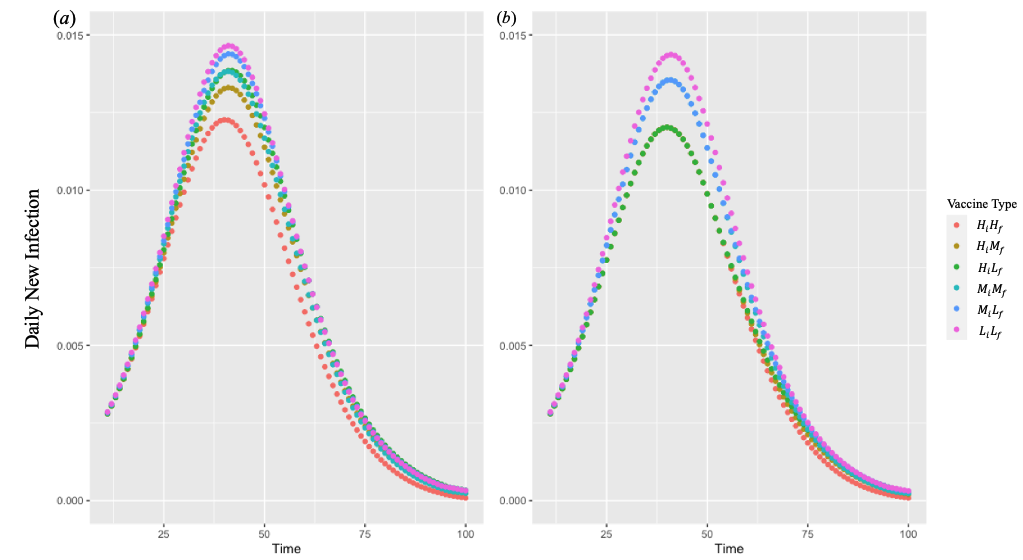
**

Figure S1: Daily new infections from day 15 to day 100 with all vaccine types when the capacity multiplier ($\lambda$) is 3 and the mutation time is on (a) day 10 and (b) day 50

1. **Main Model – Sensitivity Analyses**
   1. **Mutation Time**

Table S1 and Table S2 show the full results of the simulation under different vaccine types and capacity multipliers ($\lambda$) with a specified mutation time when the main outcome of interest is either the infection attack rate (IAR) or the mortality rate, respectively. The color scale for each mutation time shows the comparative level of IAR (green = low, red = high).

| Capacity  Multiplier ($\lambda$) | Mutation Time = Day 5 | | | | | |  | Mutation Time = Day 10 | | | | | |
| --- | --- | --- | --- | --- | --- | --- | --- | --- | --- | --- | --- | --- | --- |
|  | $H_{i}H_{f}$ | $H_{i}M_{f}$ | $H_{i}L_{f}$ | $M_{i}M_{f}$ | $M_{i}L_{f}$ | $L_{i}L_{f}$ |  | $H_{i}H_{f}$ | $H_{i}M_{f}$ | $H_{i}L_{f}$ | $M_{i}M_{f}$ | $M_{i}L_{f}$ | $L_{i}L_{f}$ |
| 3 | 56.91 | 62.77 | 65.86 | 63.44 | 66.53 | 66.86 |  | 56.75 | 61.95 | 64.72 | 63.28 | 66.04 | 66.70 |
| 2.8 | 58.82 | 64.37 | 67.29 | 65.00 | 67.90 | 68.21 |  | 58.67 | 63.61 | 66.23 | 64.85 | 67.45 | 68.06 |
| 2.6 | 60.77 | 66.00 | 68.73 | 66.57 | 69.30 | 69.58 |  | 60.63 | 65.30 | 67.76 | 66.43 | 68.88 | 69.44 |
| 2.4 | 62.75 | 67.65 | 70.19 | 68.17 | 70.71 | 70.97 |  | 62.62 | 67.01 | 69.30 | 68.05 | 70.33 | 70.84 |
| 2.2 | 64.77 | 69.32 | 71.67 | 69.79 | 72.14 | 72.38 |  | 64.65 | 68.73 | 70.86 | 69.68 | 71.79 | 72.26 |
| 2 | 66.82 | 71.01 | 73.16 | 71.44 | 73.59 | 73.80 |  | 66.71 | 70.48 | 72.43 | 71.33 | 73.28 | 73.70 |
| 1.8 | 68.90 | 72.72 | 74.67 | 73.10 | 75.05 | 75.24 |  | 68.81 | 72.25 | 74.02 | 73.00 | 74.77 | 75.15 |
| 1.6 | 71.01 | 74.45 | 76.20 | 74.78 | 76.53 | 76.70 |  | 70.93 | 74.03 | 75.63 | 74.70 | 76.29 | 76.62 |
| 1.4 | 73.16 | 76.19 | 77.74 | 76.48 | 78.03 | 78.17 |  | 73.08 | 75.84 | 77.25 | 76.41 | 77.82 | 78.10 |
| 1.2 | 75.33 | 77.96 | 79.30 | 78.21 | 79.54 | 79.67 |  | 75.27 | 77.66 | 78.88 | 78.15 | 79.36 | 79.61 |
| 1 | 77.53 | 79.75 | 80.87 | 79.95 | 81.07 | 81.17 |  | 77.48 | 79.50 | 80.53 | 79.90 | 80.93 | 81.13 |

| Capacity Multiplier  ($\lambda$) | Mutation Time = Day 15 | | | | | |  | Mutation Time = Day 20 | | | | | |
| --- | --- | --- | --- | --- | --- | --- | --- | --- | --- | --- | --- | --- | --- |
|  | $H_{i}H_{f}$ | $H_{i}M_{f}$ | $H_{i}L_{f}$ | $M_{i}M_{f}$ | $M_{i}L_{f}$ | $L_{i}L_{f}$ |  | $H_{i}H_{f}$ | $H_{i}M_{f}$ | $H_{i}L_{f}$ | $M_{i}M_{f}$ | $M_{i}L_{f}$ | $L_{i}L_{f}$ |
| 3 | 56.59 | 61.16 | 63.61 | 63.12 | 65.57 | 66.54 |  | 56.44 | 60.40 | 62.54 | 62.97 | 65.11 | 66.39 |
| 2.8 | 58.52 | 62.88 | 65.20 | 64.70 | 67.01 | 67.92 |  | 58.38 | 62.17 | 64.21 | 64.56 | 66.59 | 67.77 |
| 2.6 | 60.49 | 64.62 | 66.81 | 66.30 | 68.48 | 69.31 |  | 60.36 | 63.96 | 65.89 | 66.17 | 68.08 | 69.18 |
| 2.4 | 62.50 | 66.38 | 68.43 | 67.92 | 69.96 | 70.72 |  | 62.38 | 65.78 | 67.58 | 67.80 | 69.60 | 70.60 |
| 2.2 | 64.54 | 68.16 | 70.07 | 69.56 | 71.46 | 72.15 |  | 64.43 | 67.61 | 69.29 | 69.45 | 71.13 | 72.04 |
| 2 | 66.61 | 69.97 | 71.72 | 71.23 | 72.97 | 73.59 |  | 66.51 | 69.47 | 71.02 | 71.13 | 72.67 | 73.49 |
| 1.8 | 68.71 | 71.79 | 73.38 | 72.91 | 74.50 | 75.06 |  | 68.62 | 71.34 | 72.76 | 72.82 | 74.23 | 74.97 |
| 1.6 | 70.85 | 73.63 | 75.06 | 74.62 | 76.05 | 76.54 |  | 70.77 | 73.23 | 74.51 | 74.54 | 75.81 | 76.46 |
| 1.4 | 73.01 | 75.49 | 76.76 | 76.34 | 77.61 | 78.03 |  | 72.94 | 75.14 | 76.28 | 76.27 | 77.40 | 77.96 |
| 1.2 | 75.21 | 77.36 | 78.46 | 78.09 | 79.19 | 79.55 |  | 75.15 | 77.07 | 78.05 | 78.03 | 79.01 | 79.49 |
| 1 | 77.43 | 79.25 | 80.18 | 79.85 | 80.78 | 81.08 |  | 77.38 | 79.01 | 79.84 | 79.80 | 80.63 | 81.03 |

| Capacity Multiplier  ($\lambda$) | Mutation Time = Day 25 | | | | | |  | Mutation Time = Day 30 | | | | | |
| --- | --- | --- | --- | --- | --- | --- | --- | --- | --- | --- | --- | --- | --- |
|  | $H_{i}H_{f}$ | $H_{i}M_{f}$ | $H_{i}L_{f}$ | $M_{i}M_{f}$ | $M_{i}L_{f}$ | $L_{i}L_{f}$ |  | $H_{i}H_{f}$ | $H_{i}M_{f}$ | $H_{i}L_{f}$ | $M_{i}M_{f}$ | $M_{i}L_{f}$ | $L_{i}L_{f}$ |
| 3 | 56.30 | 59.68 | 61.52 | 62.82 | 64.67 | 66.24 |  | 56.17 | 59.00 | 60.56 | 62.69 | 64.26 | 66.10 |
| 2.8 | 58.25 | 61.50 | 63.25 | 64.42 | 66.18 | 67.64 |  | 58.13 | 60.86 | 62.35 | 64.29 | 65.79 | 67.51 |
| 2.6 | 60.24 | 63.33 | 65.00 | 66.04 | 67.71 | 69.05 |  | 60.12 | 62.74 | 64.16 | 65.92 | 67.34 | 68.93 |
| 2.4 | 62.26 | 65.20 | 66.77 | 67.68 | 69.25 | 70.48 |  | 62.15 | 64.65 | 65.99 | 67.57 | 68.91 | 70.37 |
| 2.2 | 64.32 | 67.08 | 68.55 | 69.35 | 70.81 | 71.93 |  | 64.22 | 66.57 | 67.83 | 69.25 | 70.50 | 71.83 |
| 2 | 66.41 | 68.98 | 70.34 | 71.03 | 72.38 | 73.40 |  | 66.32 | 68.52 | 69.69 | 70.94 | 72.10 | 73.30 |
| 1.8 | 68.54 | 70.91 | 72.15 | 72.74 | 73.97 | 74.88 |  | 68.46 | 70.49 | 71.56 | 72.65 | 73.72 | 74.80 |
| 1.6 | 70.69 | 72.85 | 73.97 | 74.46 | 75.58 | 76.38 |  | 70.62 | 72.48 | 73.45 | 74.39 | 75.36 | 76.31 |
| 1.4 | 72.88 | 74.81 | 75.81 | 76.21 | 77.20 | 77.90 |  | 72.81 | 74.48 | 75.35 | 76.14 | 77.01 | 77.83 |
| 1.2 | 75.09 | 76.78 | 77.65 | 77.97 | 78.84 | 79.43 |  | 75.03 | 76.50 | 77.26 | 77.92 | 78.67 | 79.38 |
| 1 | 77.33 | 78.77 | 79.51 | 79.76 | 80.49 | 80.98 |  | 77.28 | 78.54 | 79.19 | 79.71 | 80.35 | 80.93 |

| Capacity Multiplier  ($\lambda$) | Mutation Time = Day 35 | | | | | |  | Mutation Time = Day 40 | | | | | |
| --- | --- | --- | --- | --- | --- | --- | --- | --- | --- | --- | --- | --- | --- |
|  | $H_{i}H_{f}$ | $H_{i}M_{f}$ | $H_{i}L_{f}$ | $M_{i}M_{f}$ | $M_{i}L_{f}$ | $L_{i}L_{f}$ |  | $H_{i}H_{f}$ | $H_{i}M_{f}$ | $H_{i}L_{f}$ | $M_{i}M_{f}$ | $M_{i}L_{f}$ | $L_{i}L_{f}$ |
| 3 | 56.05 | 58.38 | 59.66 | 62.56 | 63.87 | 65.97 |  | 55.95 | 57.81 | 58.85 | 62.45 | 63.51 | 65.85 |
| 2.8 | 58.01 | 60.27 | 61.51 | 64.18 | 65.43 | 67.39 |  | 57.91 | 59.73 | 60.74 | 64.07 | 65.09 | 67.27 |
| 2.6 | 60.02 | 62.19 | 63.37 | 65.81 | 67.00 | 68.82 |  | 59.92 | 61.68 | 62.65 | 65.71 | 66.69 | 68.71 |
| 2.4 | 62.05 | 64.13 | 65.25 | 67.47 | 68.60 | 70.27 |  | 61.96 | 63.66 | 64.58 | 67.37 | 68.31 | 70.17 |
| 2.2 | 64.13 | 66.10 | 67.15 | 69.15 | 70.21 | 71.73 |  | 64.04 | 65.66 | 66.53 | 69.06 | 69.94 | 71.64 |
| 2 | 66.24 | 68.08 | 69.07 | 70.85 | 71.84 | 73.22 |  | 66.16 | 67.68 | 68.50 | 70.77 | 71.59 | 73.13 |
| 1.8 | 68.38 | 70.09 | 71.01 | 72.57 | 73.48 | 74.72 |  | 68.31 | 69.73 | 70.48 | 72.50 | 73.26 | 74.64 |
| 1.6 | 70.55 | 72.12 | 72.95 | 74.32 | 75.14 | 76.24 |  | 70.48 | 71.79 | 72.49 | 74.25 | 74.94 | 76.17 |
| 1.4 | 72.75 | 74.17 | 74.92 | 76.08 | 76.82 | 77.77 |  | 72.69 | 73.88 | 74.50 | 76.02 | 76.64 | 77.71 |
| 1.2 | 74.98 | 76.24 | 76.89 | 77.86 | 78.51 | 79.32 |  | 74.93 | 75.98 | 76.54 | 77.81 | 78.36 | 79.27 |
| 1 | 77.24 | 78.32 | 78.88 | 79.66 | 80.22 | 80.89 |  | 77.20 | 78.11 | 78.58 | 79.62 | 80.09 | 80.85 |

| Capacity Multiplier  ($\lambda$) | Mutation Time = Day 45 | | | | | |  | Mutation Time = Day 50 | | | | | |
| --- | --- | --- | --- | --- | --- | --- | --- | --- | --- | --- | --- | --- | --- |
|  | $H_{i}H_{f}$ | $H_{i}M_{f}$ | $H_{i}L_{f}$ | $M_{i}M_{f}$ | $M_{i}L_{f}$ | $L_{i}L_{f}$ |  | $H_{i}H_{f}$ | $H_{i}M_{f}$ | $H_{i}L_{f}$ | $M_{i}M_{f}$ | $M_{i}L_{f}$ | $L_{i}L_{f}$ |
| 3 | 55.85 | 57.31 | 58.13 | 62.34 | 63.19 | 65.75 |  | 55.77 | 56.89 | 57.51 | 62.25 | 62.91 | 65.65 |
| 2.8 | 57.82 | 59.26 | 60.05 | 63.97 | 64.79 | 67.17 |  | 57.75 | 58.85 | 59.46 | 63.88 | 64.52 | 67.08 |
| 2.6 | 59.83 | 61.23 | 62.00 | 65.62 | 66.40 | 68.62 |  | 59.76 | 60.84 | 61.43 | 65.54 | 66.15 | 68.53 |
| 2.4 | 61.88 | 63.23 | 63.97 | 67.29 | 68.04 | 70.08 |  | 61.81 | 62.86 | 63.44 | 67.21 | 67.80 | 70.00 |
| 2.2 | 63.97 | 65.26 | 65.96 | 68.98 | 69.69 | 71.56 |  | 63.90 | 64.91 | 65.46 | 68.91 | 69.47 | 71.49 |
| 2 | 66.09 | 67.31 | 67.97 | 70.70 | 71.36 | 73.06 |  | 66.03 | 66.99 | 67.51 | 70.63 | 71.16 | 72.99 |
| 1.8 | 68.24 | 69.39 | 70.01 | 72.43 | 73.05 | 74.57 |  | 68.18 | 69.09 | 69.58 | 72.37 | 72.87 | 74.51 |
| 1.6 | 70.43 | 71.49 | 72.06 | 74.19 | 74.76 | 76.11 |  | 70.37 | 71.22 | 71.68 | 74.14 | 74.59 | 76.05 |
| 1.4 | 72.64 | 73.61 | 74.13 | 75.97 | 76.48 | 77.66 |  | 72.59 | 73.37 | 73.78 | 75.92 | 76.33 | 77.61 |
| 1.2 | 74.89 | 75.75 | 76.21 | 77.77 | 78.22 | 79.22 |  | 74.85 | 75.54 | 75.91 | 77.72 | 78.09 | 79.18 |
| 1 | 77.16 | 77.91 | 78.30 | 79.58 | 79.97 | 80.81 |  | 77.13 | 77.73 | 78.05 | 79.55 | 79.87 | 80.77 |

| Capacity Multiplier  ($\lambda$) | Mutation Time = Day 55 | | | | | |  | Mutation Time = Day 60 | | | | | |
| --- | --- | --- | --- | --- | --- | --- | --- | --- | --- | --- | --- | --- | --- |
|  | $H_{i}H_{f}$ | $H_{i}M_{f}$ | $H_{i}L_{f}$ | $M_{i}M_{f}$ | $M_{i}L_{f}$ | $L_{i}L_{f}$ |  | $H_{i}H_{f}$ | $H_{i}M_{f}$ | $H_{i}L_{f}$ | $M_{i}M_{f}$ | $M_{i}L_{f}$ | $L_{i}L_{f}$ |
| 3 | 55.71 | 56.54 | 57.00 | 62.18 | 62.68 | 65.57 |  | 55.66 | 56.26 | 56.59 | 62.11 | 62.48 | 65.50 |
| 2.8 | 57.68 | 58.51 | 58.96 | 63.81 | 64.30 | 67.00 |  | 57.63 | 58.23 | 58.56 | 63.75 | 64.11 | 66.94 |
| 2.6 | 59.70 | 60.51 | 60.96 | 65.47 | 65.94 | 68.46 |  | 59.65 | 60.25 | 60.57 | 65.41 | 65.76 | 68.40 |
| 2.4 | 61.75 | 62.55 | 62.99 | 67.15 | 67.60 | 69.93 |  | 61.71 | 62.29 | 62.62 | 67.09 | 67.44 | 69.87 |
| 2.2 | 63.85 | 64.62 | 65.04 | 68.85 | 69.29 | 71.42 |  | 63.80 | 64.38 | 64.69 | 68.80 | 69.13 | 71.37 |
| 2 | 65.97 | 66.72 | 67.12 | 70.57 | 70.99 | 72.93 |  | 65.93 | 66.49 | 66.79 | 70.53 | 70.84 | 72.88 |
| 1.8 | 68.14 | 68.84 | 69.22 | 72.32 | 72.71 | 74.46 |  | 68.09 | 68.63 | 68.92 | 72.28 | 72.57 | 74.41 |
| 1.6 | 70.33 | 70.99 | 71.34 | 74.09 | 74.45 | 76.00 |  | 70.29 | 70.80 | 71.07 | 74.05 | 74.33 | 75.96 |
| 1.4 | 72.55 | 73.16 | 73.49 | 75.88 | 76.21 | 77.57 |  | 72.52 | 72.99 | 73.24 | 75.84 | 76.10 | 77.53 |
| 1.2 | 74.81 | 75.36 | 75.65 | 77.69 | 77.98 | 79.14 |  | 74.78 | 75.21 | 75.43 | 77.66 | 77.88 | 79.11 |
| 1 | 77.09 | 77.58 | 77.83 | 79.52 | 79.77 | 80.74 |  | 77.07 | 77.45 | 77.64 | 79.49 | 79.69 | 80.71 |

Table S1: Main model simulation results when the objective is to minimize infection attack rate (IAR) under different vaccine types, capacity multipliers ($\lambda$), and mutation times

| Capacity  multiplier ($\lambda$) | Mutation Time = Day 5 | | | | | |  | Mutation Time = Day 10 | | | | | |
| --- | --- | --- | --- | --- | --- | --- | --- | --- | --- | --- | --- | --- | --- |
|  | $H_{i}H_{f}$ | $H_{i}M_{f}$ | $H_{i}L_{f}$ | $M_{i}M_{f}$ | $M_{i}L_{f}$ | $L_{i}L_{f}$ |  | $H_{i}H_{f}$ | $H_{i}M_{f}$ | $H_{i}L_{f}$ | $M_{i}M_{f}$ | $M_{i}L_{f}$ | $L_{i}L_{f}$ |
| 3 | 0.8488 | 0.9331 | 0.9777 | 0.9429 | 0.9873 | 0.9921 |  | 0.8465 | 0.9214 | 0.9613 | 0.9405 | 0.9803 | 0.9897 |
| 2.8 | 0.8763 | 0.9563 | 0.9982 | 0.9653 | 1.0071 | 1.0115 |  | 0.8741 | 0.9453 | 0.9830 | 0.9631 | 1.0006 | 1.0094 |
| 2.6 | 0.9044 | 0.9797 | 1.0190 | 0.9880 | 1.0272 | 1.0313 |  | 0.9023 | 0.9696 | 1.0050 | 0.9860 | 1.0212 | 1.0293 |
| 2.4 | 0.9329 | 1.0034 | 1.0400 | 1.0110 | 1.0475 | 1.0513 |  | 0.9311 | 0.9942 | 1.0272 | 1.0091 | 1.0420 | 1.0494 |
| 2.2 | 0.9620 | 1.0274 | 1.0613 | 1.0343 | 1.0681 | 1.0715 |  | 0.9603 | 1.0190 | 1.0497 | 1.0326 | 1.0631 | 1.0698 |
| 2 | 0.9915 | 1.0518 | 1.0828 | 1.0580 | 1.0889 | 1.0920 |  | 0.9900 | 1.0442 | 1.0723 | 1.0564 | 1.0844 | 1.0905 |
| 1.8 | 1.0215 | 1.0764 | 1.1046 | 1.0819 | 1.1100 | 1.1127 |  | 1.0201 | 1.0696 | 1.0952 | 1.0805 | 1.1060 | 1.1114 |
| 1.6 | 1.0519 | 1.1013 | 1.1266 | 1.1061 | 1.1313 | 1.1337 |  | 1.0507 | 1.0953 | 1.1183 | 1.1049 | 1.1278 | 1.1325 |
| 1.4 | 1.0827 | 1.1265 | 1.1488 | 1.1306 | 1.1529 | 1.1550 |  | 1.0817 | 1.1213 | 1.1416 | 1.1296 | 1.1498 | 1.1539 |
| 1.2 | 1.1140 | 1.1519 | 1.1712 | 1.1555 | 1.1747 | 1.1764 |  | 1.1131 | 1.1476 | 1.1651 | 1.1546 | 1.1721 | 1.1756 |
| 1 | 1.1457 | 1.1776 | 1.1938 | 1.1805 | 1.1967 | 1.1982 |  | 1.1449 | 1.1740 | 1.1888 | 1.1798 | 1.1946 | 1.1975 |

| Capacity multiplier ($\lambda$) | Mutation Time = Day 15 | | | | | |  | Mutation Time = Day 20 | | | | | |
| --- | --- | --- | --- | --- | --- | --- | --- | --- | --- | --- | --- | --- | --- |
|  | $H_{i}H_{f}$ | $H_{i}M_{f}$ | $H_{i}L_{f}$ | $M_{i}M_{f}$ | $M_{i}L_{f}$ | $L_{i}L_{f}$ |  | $H_{i}H_{f}$ | $H_{i}M_{f}$ | $H_{i}L_{f}$ | $M_{i}M_{f}$ | $M_{i}L_{f}$ | $L_{i}L_{f}$ |
| 3 | 0.8442 | 0.9100 | 0.9453 | 0.9382 | 0.9735 | 0.9875 |  | 0.8421 | 0.8991 | 0.9299 | 0.9361 | 0.9669 | 0.9853 |
| 2.8 | 0.8720 | 0.9347 | 0.9682 | 0.9610 | 0.9943 | 1.0073 |  | 0.8700 | 0.9245 | 0.9539 | 0.9589 | 0.9882 | 1.0052 |
| 2.6 | 0.9004 | 0.9598 | 0.9913 | 0.9840 | 1.0154 | 1.0273 |  | 0.8985 | 0.9504 | 0.9781 | 0.9821 | 1.0097 | 1.0254 |
| 2.4 | 0.9293 | 0.9852 | 1.0147 | 1.0074 | 1.0367 | 1.0476 |  | 0.9275 | 0.9765 | 1.0025 | 1.0056 | 1.0315 | 1.0459 |
| 2.2 | 0.9586 | 1.0108 | 1.0382 | 1.0310 | 1.0582 | 1.0682 |  | 0.9570 | 1.0029 | 1.0271 | 1.0294 | 1.0535 | 1.0666 |
| 2 | 0.9885 | 1.0368 | 1.0620 | 1.0550 | 1.0800 | 1.0890 |  | 0.9870 | 1.0296 | 1.0519 | 1.0535 | 1.0757 | 1.0876 |
| 1.8 | 1.0188 | 1.0630 | 1.0860 | 1.0792 | 1.1021 | 1.1101 |  | 1.0175 | 1.0566 | 1.0770 | 1.0779 | 1.0982 | 1.1088 |
| 1.6 | 1.0495 | 1.0895 | 1.1102 | 1.1038 | 1.1243 | 1.1314 |  | 1.0484 | 1.0838 | 1.1022 | 1.1026 | 1.1209 | 1.1303 |
| 1.4 | 1.0807 | 1.1163 | 1.1346 | 1.1286 | 1.1468 | 1.1529 |  | 1.0797 | 1.1113 | 1.1277 | 1.1276 | 1.1439 | 1.1520 |
| 1.2 | 1.1122 | 1.1433 | 1.1591 | 1.1537 | 1.1695 | 1.1747 |  | 1.1114 | 1.1390 | 1.1533 | 1.1529 | 1.1670 | 1.1739 |
| 1 | 1.1442 | 1.1705 | 1.1839 | 1.1791 | 1.1925 | 1.1968 |  | 1.1435 | 1.1670 | 1.1790 | 1.1784 | 1.1904 | 1.1961 |

| Capacity multiplier ($\lambda$) | Mutation Time = Day 25 | | | | | |  | Mutation Time = Day 30 | | | | | |
| --- | --- | --- | --- | --- | --- | --- | --- | --- | --- | --- | --- | --- | --- |
|  | $H_{i}H_{f}$ | $H_{i}M_{f}$ | $H_{i}L_{f}$ | $M_{i}M_{f}$ | $M_{i}L_{f}$ | $L_{i}L_{f}$ |  | $H_{i}H_{f}$ | $H_{i}M_{f}$ | $H_{i}L_{f}$ | $M_{i}M_{f}$ | $M_{i}L_{f}$ | $L_{i}L_{f}$ |
| 3 | 0.8401 | 0.8887 | 0.9152 | 0.9340 | 0.9606 | 0.9832 |  | 0.8382 | 0.8789 | 0.9014 | 0.9320 | 0.9546 | 0.9812 |
| 2.8 | 0.8681 | 0.9148 | 0.9402 | 0.9570 | 0.9823 | 1.0033 |  | 0.8664 | 0.9057 | 0.9271 | 0.9551 | 0.9767 | 1.0014 |
| 2.6 | 0.8967 | 0.9413 | 0.9653 | 0.9803 | 1.0042 | 1.0236 |  | 0.8951 | 0.9328 | 0.9532 | 0.9786 | 0.9990 | 1.0219 |
| 2.4 | 0.9259 | 0.9681 | 0.9907 | 1.0040 | 1.0264 | 1.0442 |  | 0.9243 | 0.9602 | 0.9795 | 1.0024 | 1.0216 | 1.0426 |
| 2.2 | 0.9555 | 0.9952 | 1.0164 | 1.0279 | 1.0489 | 1.0651 |  | 0.9541 | 0.9879 | 1.0060 | 1.0264 | 1.0445 | 1.0636 |
| 2 | 0.9856 | 1.0226 | 1.0422 | 1.0521 | 1.0716 | 1.0862 |  | 0.9843 | 1.0160 | 1.0328 | 1.0508 | 1.0676 | 1.0849 |
| 1.8 | 1.0162 | 1.0503 | 1.0682 | 1.0767 | 1.0945 | 1.1076 |  | 1.0150 | 1.0443 | 1.0598 | 1.0755 | 1.0909 | 1.1064 |
| 1.6 | 1.0473 | 1.0783 | 1.0945 | 1.1015 | 1.1176 | 1.1292 |  | 1.0462 | 1.0729 | 1.0870 | 1.1005 | 1.1144 | 1.1281 |
| 1.4 | 1.0787 | 1.1065 | 1.1209 | 1.1267 | 1.1410 | 1.1510 |  | 1.0778 | 1.1018 | 1.1143 | 1.1257 | 1.1382 | 1.1501 |
| 1.2 | 1.1106 | 1.1349 | 1.1475 | 1.1521 | 1.1646 | 1.1731 |  | 1.1098 | 1.1309 | 1.1419 | 1.1513 | 1.1622 | 1.1723 |
| 1 | 1.1428 | 1.1636 | 1.1742 | 1.1777 | 1.1883 | 1.1954 |  | 1.1422 | 1.1602 | 1.1696 | 1.1771 | 1.1863 | 1.1947 |

| Capacity multiplier ($\lambda$) | Mutation Time = Day 35 | | | | | |  | Mutation Time = Day 40 | | | | | |
| --- | --- | --- | --- | --- | --- | --- | --- | --- | --- | --- | --- | --- | --- |
|  | $H_{i}H_{f}$ | $H_{i}M_{f}$ | $H_{i}L_{f}$ | $M_{i}M_{f}$ | $M_{i}L_{f}$ | $L_{i}L_{f}$ |  | $H_{i}H_{f}$ | $H_{i}M_{f}$ | $H_{i}L_{f}$ | $M_{i}M_{f}$ | $M_{i}L_{f}$ | $L_{i}L_{f}$ |
| 3 | 0.8365 | 0.8700 | 0.8885 | 0.9302 | 0.9490 | 0.9793 |  | 0.8349 | 0.8618 | 0.8768 | 0.9285 | 0.9439 | 0.9776 |
| 2.8 | 0.8647 | 0.8972 | 0.9150 | 0.9534 | 0.9714 | 0.9997 |  | 0.8633 | 0.8895 | 0.9039 | 0.9518 | 0.9666 | 0.9980 |
| 2.6 | 0.8935 | 0.9248 | 0.9418 | 0.9770 | 0.9941 | 1.0203 |  | 0.8922 | 0.9175 | 0.9314 | 0.9755 | 0.9896 | 1.0187 |
| 2.4 | 0.9229 | 0.9528 | 0.9689 | 1.0009 | 1.0171 | 1.0411 |  | 0.9216 | 0.9459 | 0.9592 | 0.9995 | 1.0129 | 1.0397 |
| 2.2 | 0.9528 | 0.9811 | 0.9963 | 1.0251 | 1.0403 | 1.0622 |  | 0.9515 | 0.9747 | 0.9873 | 1.0238 | 1.0364 | 1.0609 |
| 2 | 0.9831 | 1.0097 | 1.0239 | 1.0496 | 1.0637 | 1.0836 |  | 0.9820 | 1.0039 | 1.0157 | 1.0484 | 1.0602 | 1.0824 |
| 1.8 | 1.0139 | 1.0386 | 1.0518 | 1.0744 | 1.0874 | 1.1052 |  | 1.0129 | 1.0333 | 1.0443 | 1.0733 | 1.0842 | 1.1041 |
| 1.6 | 1.0452 | 1.0679 | 1.0798 | 1.0994 | 1.1113 | 1.1271 |  | 1.0443 | 1.0631 | 1.0731 | 1.0985 | 1.1085 | 1.1261 |
| 1.4 | 1.0769 | 1.0973 | 1.1081 | 1.1248 | 1.1355 | 1.1492 |  | 1.0761 | 1.0931 | 1.1021 | 1.1240 | 1.1330 | 1.1483 |
| 1.2 | 1.1090 | 1.1271 | 1.1365 | 1.1505 | 1.1599 | 1.1715 |  | 1.1083 | 1.1235 | 1.1314 | 1.1498 | 1.1577 | 1.1708 |
| 1 | 1.1415 | 1.1570 | 1.1651 | 1.1764 | 1.1844 | 1.1941 |  | 1.1409 | 1.1540 | 1.1608 | 1.1758 | 1.1826 | 1.1935 |

| Capacity multiplier ($\lambda$) | Mutation Time = Day 45 | | | | | |  | Mutation Time = Day 50 | | | | | |
| --- | --- | --- | --- | --- | --- | --- | --- | --- | --- | --- | --- | --- | --- |
|  | $H_{i}H_{f}$ | $H_{i}M_{f}$ | $H_{i}L_{f}$ | $M_{i}M_{f}$ | $M_{i}L_{f}$ | $L_{i}L_{f}$ |  | $H_{i}H_{f}$ | $H_{i}M_{f}$ | $H_{i}L_{f}$ | $M_{i}M_{f}$ | $M_{i}L_{f}$ | $L_{i}L_{f}$ |
| 3 | 0.8336 | 0.8547 | 0.8664 | 0.9270 | 0.9393 | 0.9760 |  | 0.8325 | 0.8485 | 0.8575 | 0.9257 | 0.9352 | 0.9747 |
| 2.8 | 0.8620 | 0.8826 | 0.8940 | 0.9504 | 0.9622 | 0.9966 |  | 0.8609 | 0.8767 | 0.8855 | 0.9492 | 0.9584 | 0.9953 |
| 2.6 | 0.8909 | 0.9110 | 0.9221 | 0.9742 | 0.9855 | 1.0174 |  | 0.8899 | 0.9054 | 0.9139 | 0.9730 | 0.9819 | 1.0161 |
| 2.4 | 0.9204 | 0.9398 | 0.9504 | 0.9982 | 1.0091 | 1.0384 |  | 0.9194 | 0.9345 | 0.9428 | 0.9971 | 1.0057 | 1.0373 |
| 2.2 | 0.9504 | 0.9690 | 0.9791 | 1.0226 | 1.0329 | 1.0597 |  | 0.9495 | 0.9640 | 0.9720 | 1.0216 | 1.0297 | 1.0587 |
| 2 | 0.9810 | 0.9986 | 1.0081 | 1.0473 | 1.0569 | 1.0813 |  | 0.9801 | 0.9940 | 1.0015 | 1.0464 | 1.0540 | 1.0803 |
| 1.8 | 1.0120 | 1.0285 | 1.0374 | 1.0723 | 1.0812 | 1.1031 |  | 1.0111 | 1.0243 | 1.0313 | 1.0715 | 1.0786 | 1.1023 |
| 1.6 | 1.0434 | 1.0588 | 1.0669 | 1.0976 | 1.1058 | 1.1252 |  | 1.0427 | 1.0549 | 1.0614 | 1.0968 | 1.1034 | 1.1244 |
| 1.4 | 1.0753 | 1.0893 | 1.0967 | 1.1232 | 1.1306 | 1.1475 |  | 1.0746 | 1.0858 | 1.0918 | 1.1225 | 1.1285 | 1.1468 |
| 1.2 | 1.1076 | 1.1201 | 1.1267 | 1.1491 | 1.1556 | 1.1701 |  | 1.1071 | 1.1171 | 1.1224 | 1.1485 | 1.1538 | 1.1695 |
| 1 | 1.1404 | 1.1512 | 1.1568 | 1.1753 | 1.1809 | 1.1929 |  | 1.1399 | 1.1486 | 1.1532 | 1.1748 | 1.1793 | 1.1924 |

| Capacity multiplier ($\lambda$) | Mutation Time = Day 55 | | | | | |  | Mutation Time = Day 60 | | | | | |
| --- | --- | --- | --- | --- | --- | --- | --- | --- | --- | --- | --- | --- | --- |
|  | $H_{i}H_{f}$ | $H_{i}M_{f}$ | $H_{i}L_{f}$ | $M_{i}M_{f}$ | $M_{i}L_{f}$ | $L_{i}L_{f}$ |  | $H_{i}H_{f}$ | $H_{i}M_{f}$ | $H_{i}L_{f}$ | $M_{i}M_{f}$ | $M_{i}L_{f}$ | $L_{i}L_{f}$ |
| 3 | 0.8315 | 0.8435 | 0.8501 | 0.9246 | 0.9318 | 0.9735 |  | 0.8308 | 0.8394 | 0.8442 | 0.9237 | 0.9290 | 0.9725 |
| 2.8 | 0.8600 | 0.8718 | 0.8784 | 0.9481 | 0.9552 | 0.9942 |  | 0.8592 | 0.8679 | 0.8726 | 0.9473 | 0.9525 | 0.9932 |
| 2.6 | 0.8890 | 0.9007 | 0.9071 | 0.9720 | 0.9788 | 1.0151 |  | 0.8883 | 0.8969 | 0.9016 | 0.9712 | 0.9763 | 1.0142 |
| 2.4 | 0.9186 | 0.9300 | 0.9363 | 0.9962 | 1.0028 | 1.0363 |  | 0.9179 | 0.9263 | 0.9310 | 0.9954 | 1.0004 | 1.0355 |
| 2.2 | 0.9487 | 0.9598 | 0.9659 | 1.0207 | 1.0270 | 1.0578 |  | 0.9480 | 0.9563 | 0.9608 | 1.0200 | 1.0247 | 1.0570 |
| 2 | 0.9793 | 0.9900 | 0.9958 | 1.0456 | 1.0515 | 1.0795 |  | 0.9787 | 0.9867 | 0.9911 | 1.0449 | 1.0494 | 1.0788 |
| 1.8 | 1.0104 | 1.0206 | 1.0260 | 1.0707 | 1.0763 | 1.1015 |  | 1.0099 | 1.0175 | 1.0217 | 1.0701 | 1.0744 | 1.1008 |
| 1.6 | 1.0420 | 1.0515 | 1.0566 | 1.0962 | 1.1013 | 1.1237 |  | 1.0415 | 1.0487 | 1.0526 | 1.0956 | 1.0996 | 1.1231 |
| 1.4 | 1.0741 | 1.0828 | 1.0875 | 1.1219 | 1.1266 | 1.1462 |  | 1.0736 | 1.0803 | 1.0839 | 1.1214 | 1.1251 | 1.1457 |
| 1.2 | 1.1065 | 1.1145 | 1.1187 | 1.1480 | 1.1522 | 1.1689 |  | 1.1061 | 1.1122 | 1.1155 | 1.1475 | 1.1508 | 1.1685 |
| 1 | 1.1394 | 1.1464 | 1.1500 | 1.1743 | 1.1780 | 1.1919 |  | 1.1391 | 1.1445 | 1.1473 | 1.1739 | 1.1768 | 1.1915 |

Table S2: Main model simulation results when the objective is to minimize the mortality rate under different vaccine types, capacity multipliers ($\lambda$), and mutation times.

- 1. **Time-to-immunity: the amount of time for individuals to build immunity after vaccination**

After vaccination, it may take several days for individuals to build immunity and become protected from the disease.^1^ We ran simulations where individuals build immunity 7, 14, and 21 days after their vaccinations, i.e., entering the ­*Vaccinated with immunity* compartment ($V$). Table S3 and Table S4 show the infection attack rate (IAR) and the mortality rate, respectively, under different vaccine types and capacity multipliers ($\lambda$) when the mutation time is Day 10, Day 30, and Day 50. While the findings were consistent with the main model (i.e., time-to-immunity = 0 days), we observed that both the IAR and mortality rate increase as the time-to-immunity becomes larger, i.e., building immunization is delayed, as more vaccinated individuals become infected before they can build immunity even if their vaccines are effective.

| $\lambda$ | Time-to-immunity = 7 days | | | | | | | | | | | | | | | | | | | |
| --- | --- | --- | --- | --- | --- | --- | --- | --- | --- | --- | --- | --- | --- | --- | --- | --- | --- | --- | --- | --- |
|  | Mutation Time = Day 10 | | | | | |  | Mutation Time = Day 30 | | | | | |  | Mutation Time = Day 50 | | | | | |
|  | $H_{i}H_{f}$ | $H_{i}M_{f}$ | $H_{i}L_{f}$ | $M_{i}M_{f}$ | $M_{i}L_{f}$ | $L_{i}L_{f}$ |  | $H_{i}H_{f}$ | $H_{i}M_{f}$ | $H_{i}L_{f}$ | $M_{i}M_{f}$ | $M_{i}L_{f}$ | $L_{i}L_{f}$ |  | $H_{i}H_{f}$ | $H_{i}M_{f}$ | $H_{i}L_{f}$ | $M_{i}M_{f}$ | $M_{i}L_{f}$ | $L_{i}L_{f}$ |
| 3 | 61.23 | 66.38 | 69.10 | 66.76 | 69.48 | 69.67 |  | 60.66 | 63.45 | 64.98 | 66.18 | 67.72 | 69.08 |  | 60.25 | 61.34 | 61.93 | 65.74 | 66.38 | 68.63 |
| 2.8 | 62.84 | 67.72 | 70.29 | 68.08 | 70.65 | 70.82 |  | 62.30 | 64.99 | 66.45 | 67.53 | 69.00 | 70.27 |  | 61.92 | 62.98 | 63.57 | 67.12 | 67.74 | 69.85 |
| 2.6 | 64.48 | 69.09 | 71.50 | 69.42 | 71.83 | 71.99 |  | 63.98 | 66.55 | 67.93 | 68.91 | 70.30 | 71.48 |  | 63.61 | 64.65 | 65.22 | 68.52 | 69.12 | 71.09 |
| 2.4 | 66.15 | 70.47 | 72.73 | 70.78 | 73.03 | 73.18 |  | 65.69 | 68.13 | 69.44 | 70.31 | 71.62 | 72.71 |  | 65.35 | 66.36 | 66.91 | 69.95 | 70.52 | 72.34 |
| 2.2 | 67.86 | 71.89 | 73.97 | 72.16 | 74.25 | 74.38 |  | 67.44 | 69.74 | 70.97 | 71.73 | 72.96 | 73.95 |  | 67.12 | 68.09 | 68.62 | 71.40 | 71.94 | 73.61 |
| 2 | 69.61 | 73.32 | 75.24 | 73.57 | 75.48 | 75.61 |  | 69.22 | 71.38 | 72.52 | 73.18 | 74.32 | 75.22 |  | 68.93 | 69.86 | 70.36 | 72.87 | 73.39 | 74.91 |
| 1.8 | 71.39 | 74.78 | 76.52 | 75.00 | 76.74 | 76.85 |  | 71.04 | 73.03 | 74.08 | 74.65 | 75.70 | 76.51 |  | 70.77 | 71.65 | 72.12 | 74.37 | 74.85 | 76.22 |
| 1.6 | 73.20 | 76.26 | 77.83 | 76.45 | 78.02 | 78.12 |  | 72.89 | 74.71 | 75.67 | 76.14 | 77.10 | 77.81 |  | 72.65 | 73.47 | 73.91 | 75.89 | 76.34 | 77.56 |
| 1.4 | 75.04 | 77.76 | 79.15 | 77.93 | 79.32 | 79.40 |  | 74.77 | 76.41 | 77.27 | 77.66 | 78.51 | 79.14 |  | 74.56 | 75.31 | 75.71 | 77.44 | 77.84 | 78.91 |
| 1.2 | 76.92 | 79.29 | 80.49 | 79.43 | 80.64 | 80.71 |  | 76.69 | 78.14 | 78.89 | 79.20 | 79.95 | 80.48 |  | 76.51 | 77.19 | 77.54 | 79.01 | 79.37 | 80.29 |
| 1 | 78.84 | 80.84 | 81.86 | 80.96 | 81.98 | 82.03 |  | 78.65 | 79.89 | 80.52 | 80.77 | 81.41 | 81.84 |  | 78.49 | 79.08 | 79.40 | 80.61 | 80.92 | 81.68 |

| $\lambda$ | Time-to-immunity = 14 days | | | | | | | | | | | | | | | | | | | |
| --- | --- | --- | --- | --- | --- | --- | --- | --- | --- | --- | --- | --- | --- | --- | --- | --- | --- | --- | --- | --- |
|  | Mutation Time = Day 10 | | | | | |  | Mutation Time = Day 30 | | | | | |  | Mutation Time = Day 50 | | | | | |
|  | $H_{i}H_{f}$ | $H_{i}M_{f}$ | $H_{i}L_{f}$ | $M_{i}M_{f}$ | $M_{i}L_{f}$ | $L_{i}L_{f}$ |  | $H_{i}H_{f}$ | $H_{i}M_{f}$ | $H_{i}L_{f}$ | $M_{i}M_{f}$ | $M_{i}L_{f}$ | $L_{i}L_{f}$ |  | $H_{i}H_{f}$ | $H_{i}M_{f}$ | $H_{i}L_{f}$ | $M_{i}M_{f}$ | $M_{i}L_{f}$ | $L_{i}L_{f}$ |
| 3 | 65.31 | 69.94 | 72.39 | 69.94 | 72.39 | 72.39 |  | 64.86 | 67.65 | 69.16 | 69.48 | 71.00 | 71.93 |  | 64.46 | 65.52 | 66.11 | 69.05 | 69.67 | 71.48 |
| 2.8 | 66.64 | 71.04 | 73.35 | 71.04 | 73.35 | 73.35 |  | 66.22 | 68.90 | 70.34 | 70.61 | 72.06 | 72.92 |  | 65.84 | 66.88 | 67.45 | 70.20 | 70.80 | 72.49 |
| 2.6 | 68.00 | 72.15 | 74.32 | 72.15 | 74.32 | 74.32 |  | 67.61 | 70.16 | 71.53 | 71.75 | 73.13 | 73.93 |  | 67.25 | 68.27 | 68.82 | 71.37 | 71.95 | 73.53 |
| 2.4 | 69.40 | 73.29 | 75.32 | 73.29 | 75.32 | 75.32 |  | 69.03 | 71.46 | 72.75 | 72.92 | 74.22 | 74.95 |  | 68.69 | 69.68 | 70.22 | 72.56 | 73.12 | 74.59 |
| 2.2 | 70.83 | 74.45 | 76.34 | 74.45 | 76.34 | 76.34 |  | 70.49 | 72.77 | 73.98 | 74.12 | 75.33 | 76.00 |  | 70.17 | 71.13 | 71.64 | 73.78 | 74.32 | 75.66 |
| 2 | 72.29 | 75.64 | 77.38 | 75.64 | 77.38 | 77.38 |  | 71.98 | 74.11 | 75.24 | 75.33 | 76.46 | 77.07 |  | 71.69 | 72.60 | 73.09 | 75.03 | 75.53 | 76.76 |
| 1.8 | 73.78 | 76.85 | 78.44 | 76.85 | 78.44 | 78.44 |  | 73.51 | 75.48 | 76.52 | 76.58 | 77.61 | 78.16 |  | 73.24 | 74.10 | 74.56 | 76.30 | 76.77 | 77.88 |
| 1.6 | 75.31 | 78.09 | 79.52 | 78.09 | 79.52 | 79.52 |  | 75.07 | 76.87 | 77.81 | 77.84 | 78.79 | 79.27 |  | 74.83 | 75.63 | 76.06 | 77.59 | 78.03 | 79.02 |
| 1.4 | 76.88 | 79.35 | 80.62 | 79.35 | 80.62 | 80.62 |  | 76.67 | 78.29 | 79.13 | 79.14 | 79.98 | 80.41 |  | 76.45 | 77.19 | 77.58 | 78.92 | 79.32 | 80.18 |
| 1.2 | 78.48 | 80.64 | 81.74 | 80.64 | 81.74 | 81.74 |  | 78.30 | 79.73 | 80.47 | 80.46 | 81.20 | 81.56 |  | 78.12 | 78.78 | 79.13 | 80.27 | 80.62 | 81.37 |
| 1 | 80.13 | 81.96 | 82.89 | 81.96 | 82.89 | 82.89 |  | 79.98 | 81.20 | 81.83 | 81.81 | 82.44 | 82.74 |  | 79.82 | 80.40 | 80.71 | 81.65 | 81.95 | 82.58 |

| $\lambda$ | Time-to-immunity = 21 days | | | | | | | | | | | | | | | | | | | |
| --- | --- | --- | --- | --- | --- | --- | --- | --- | --- | --- | --- | --- | --- | --- | --- | --- | --- | --- | --- | --- |
|  | Mutation Time = Day 10 | | | | | |  | Mutation Time = Day 30 | | | | | |  | Mutation Time = Day 50 | | | | | |
|  | $H_{i}H_{f}$ | $H_{i}M_{f}$ | $H_{i}L_{f}$ | $M_{i}M_{f}$ | $M_{i}L_{f}$ | $L_{i}L_{f}$ |  | $H_{i}H_{f}$ | $H_{i}M_{f}$ | $H_{i}L_{f}$ | $M_{i}M_{f}$ | $M_{i}L_{f}$ | $L_{i}L_{f}$ |  | $H_{i}H_{f}$ | $H_{i}M_{f}$ | $H_{i}L_{f}$ | $M_{i}M_{f}$ | $M_{i}L_{f}$ | $L_{i}L_{f}$ |
| 3 | 69.05 | 72.86 | 74.88 | 72.86 | 74.88 | 74.88 |  | 68.81 | 71.61 | 73.12 | 72.61 | 74.13 | 74.63 |  | 68.40 | 69.46 | 70.05 | 72.17 | 72.79 | 74.18 |
| 2.8 | 70.14 | 73.76 | 75.67 | 73.76 | 75.67 | 75.67 |  | 69.91 | 72.59 | 74.03 | 73.52 | 74.97 | 75.44 |  | 69.52 | 70.56 | 71.13 | 73.11 | 73.71 | 75.01 |
| 2.6 | 71.25 | 74.68 | 76.48 | 74.68 | 76.48 | 76.48 |  | 71.04 | 73.59 | 74.96 | 74.46 | 75.83 | 76.26 |  | 70.67 | 71.68 | 72.24 | 74.07 | 74.65 | 75.87 |
| 2.4 | 72.39 | 75.62 | 77.31 | 75.62 | 77.31 | 77.31 |  | 72.20 | 74.62 | 75.90 | 75.42 | 76.71 | 77.11 |  | 71.85 | 72.83 | 73.37 | 75.06 | 75.61 | 76.74 |
| 2.2 | 73.57 | 76.58 | 78.16 | 76.58 | 78.16 | 78.16 |  | 73.39 | 75.66 | 76.87 | 76.40 | 77.60 | 77.97 |  | 73.07 | 74.01 | 74.52 | 76.06 | 76.59 | 77.63 |
| 2 | 74.78 | 77.57 | 79.02 | 77.57 | 79.02 | 79.02 |  | 74.61 | 76.73 | 77.85 | 77.40 | 78.52 | 78.86 |  | 74.32 | 75.22 | 75.70 | 77.10 | 77.59 | 78.55 |
| 1.8 | 76.02 | 78.58 | 79.91 | 78.58 | 79.91 | 79.91 |  | 75.87 | 77.83 | 78.86 | 78.43 | 79.46 | 79.76 |  | 75.60 | 76.45 | 76.91 | 78.15 | 78.62 | 79.48 |
| 1.6 | 77.29 | 79.62 | 80.83 | 79.62 | 80.83 | 80.83 |  | 77.16 | 78.95 | 79.89 | 79.49 | 80.42 | 80.69 |  | 76.92 | 77.71 | 78.14 | 79.24 | 79.67 | 80.44 |
| 1.4 | 78.61 | 80.69 | 81.76 | 80.69 | 81.76 | 81.76 |  | 78.49 | 80.10 | 80.94 | 80.57 | 81.41 | 81.64 |  | 78.28 | 79.01 | 79.40 | 80.35 | 80.74 | 81.42 |
| 1.2 | 79.96 | 81.78 | 82.72 | 81.78 | 82.72 | 82.72 |  | 79.86 | 81.28 | 82.02 | 81.68 | 82.42 | 82.62 |  | 79.67 | 80.33 | 80.68 | 81.49 | 81.84 | 82.43 |
| 1 | 81.35 | 82.90 | 83.70 | 82.90 | 83.70 | 83.70 |  | 81.27 | 82.49 | 83.11 | 82.82 | 83.45 | 83.61 |  | 81.11 | 81.69 | 81.99 | 82.66 | 82.97 | 83.45 |

Table S3: Infection Attack Rate (IAR) under different times-to-immunity, mutation times, and capacity multipliers for each vaccine type

| $\lambda$ | Time-to-immunity = 7 days | | | | | | | | | | | | | | | | | | | |
| --- | --- | --- | --- | --- | --- | --- | --- | --- | --- | --- | --- | --- | --- | --- | --- | --- | --- | --- | --- | --- |
|  | Mutation Time = Day 10 | | | | | |  | Mutation Time = Day 30 | | | | | |  | Mutation Time = Day 50 | | | | | |
|  | $H_{i}H_{f}$ | $H_{i}M_{f}$ | $H_{i}L_{f}$ | $M_{i}M_{f}$ | $M_{i}L_{f}$ | $L_{i}L_{f}$ |  | $H_{i}H_{f}$ | $H_{i}M_{f}$ | $H_{i}L_{f}$ | $M_{i}M_{f}$ | $M_{i}L_{f}$ | $L_{i}L_{f}$ |  | $H_{i}H_{f}$ | $H_{i}M_{f}$ | $H_{i}L_{f}$ | $M_{i}M_{f}$ | $M_{i}L_{f}$ | $L_{i}L_{f}$ |
| 3 | 0.9110 | 0.9851 | 1.0243 | 0.9907 | 1.0298 | 1.0326 |  | 0.9027 | 0.9430 | 0.9649 | 0.9823 | 1.0044 | 1.0241 |  | 0.8970 | 0.9125 | 0.9211 | 0.9760 | 0.9851 | 1.0176 |
| 2.8 | 0.9342 | 1.0045 | 1.0415 | 1.0096 | 1.0466 | 1.0491 |  | 0.9264 | 0.9651 | 0.9861 | 1.0018 | 1.0229 | 1.0412 |  | 0.9209 | 0.9362 | 0.9446 | 0.9958 | 1.0047 | 1.0351 |
| 2.6 | 0.9578 | 1.0241 | 1.0589 | 1.0289 | 1.0636 | 1.0659 |  | 0.9506 | 0.9876 | 1.0075 | 1.0216 | 1.0416 | 1.0586 |  | 0.9453 | 0.9603 | 0.9685 | 1.0160 | 1.0246 | 1.0529 |
| 2.4 | 0.9819 | 1.0441 | 1.0765 | 1.0485 | 1.0809 | 1.0830 |  | 0.9752 | 1.0104 | 1.0293 | 1.0417 | 1.0606 | 1.0763 |  | 0.9703 | 0.9849 | 0.9928 | 1.0366 | 1.0448 | 1.0710 |
| 2.2 | 1.0065 | 1.0644 | 1.0945 | 1.0684 | 1.0984 | 1.1004 |  | 1.0004 | 1.0336 | 1.0513 | 1.0622 | 1.0799 | 1.0942 |  | 0.9958 | 1.0098 | 1.0174 | 1.0574 | 1.0653 | 1.0893 |
| 2 | 1.0316 | 1.0851 | 1.1127 | 1.0886 | 1.1162 | 1.1180 |  | 1.0261 | 1.0571 | 1.0735 | 1.0831 | 1.0995 | 1.1124 |  | 1.0218 | 1.0352 | 1.0425 | 1.0786 | 1.0861 | 1.1079 |
| 1.8 | 1.0572 | 1.1060 | 1.1312 | 1.1092 | 1.1344 | 1.1359 |  | 1.0522 | 1.0809 | 1.0961 | 1.1042 | 1.1193 | 1.1309 |  | 1.0483 | 1.0610 | 1.0678 | 1.1002 | 1.1071 | 1.1269 |
| 1.6 | 1.0833 | 1.1274 | 1.1500 | 1.1302 | 1.1528 | 1.1542 |  | 1.0789 | 1.1051 | 1.1189 | 1.1257 | 1.1395 | 1.1497 |  | 1.0754 | 1.0872 | 1.0935 | 1.1221 | 1.1285 | 1.1461 |
| 1.4 | 1.1099 | 1.1490 | 1.1690 | 1.1515 | 1.1715 | 1.1727 |  | 1.1060 | 1.1296 | 1.1419 | 1.1476 | 1.1599 | 1.1688 |  | 1.1029 | 1.1138 | 1.1195 | 1.1444 | 1.1502 | 1.1656 |
| 1.2 | 1.1370 | 1.1710 | 1.1884 | 1.1731 | 1.1904 | 1.1915 |  | 1.1337 | 1.1545 | 1.1653 | 1.1698 | 1.1805 | 1.1882 |  | 1.1310 | 1.1407 | 1.1459 | 1.1670 | 1.1722 | 1.1854 |
| 1 | 1.1645 | 1.1934 | 1.2080 | 1.1951 | 1.2097 | 1.2106 |  | 1.1618 | 1.1796 | 1.1888 | 1.1923 | 1.2015 | 1.2078 |  | 1.1595 | 1.1681 | 1.1726 | 1.1900 | 1.1945 | 1.2055 |

| $\lambda$ | Time-to-immunity = 14 days | | | | | | | | | | | | | | | | | | | |
| --- | --- | --- | --- | --- | --- | --- | --- | --- | --- | --- | --- | --- | --- | --- | --- | --- | --- | --- | --- | --- |
|  | Mutation Time = Day 10 | | | | | |  | Mutation Time = Day 30 | | | | | |  | Mutation Time = Day 50 | | | | | |
|  | $H_{i}H_{f}$ | $H_{i}M_{f}$ | $H_{i}L_{f}$ | $M_{i}M_{f}$ | $M_{i}L_{f}$ | $L_{i}L_{f}$ |  | $H_{i}H_{f}$ | $H_{i}M_{f}$ | $H_{i}L_{f}$ | $M_{i}M_{f}$ | $M_{i}L_{f}$ | $L_{i}L_{f}$ |  | $H_{i}H_{f}$ | $H_{i}M_{f}$ | $H_{i}L_{f}$ | $M_{i}M_{f}$ | $M_{i}L_{f}$ | $L_{i}L_{f}$ |
| 3 | 0.9698 | 1.0365 | 1.0717 | 1.0365 | 1.0717 | 1.0717 |  | 0.9633 | 1.0035 | 1.0252 | 1.0299 | 1.0517 | 1.0650 |  | 0.9575 | 0.9728 | 0.9812 | 1.0236 | 1.0325 | 1.0586 |
| 2.8 | 0.9890 | 1.0522 | 1.0855 | 1.0522 | 1.0855 | 1.0855 |  | 0.9829 | 1.0214 | 1.0421 | 1.0460 | 1.0669 | 1.0793 |  | 0.9773 | 0.9924 | 1.0006 | 1.0401 | 1.0488 | 1.0732 |
| 2.6 | 1.0086 | 1.0683 | 1.0996 | 1.0683 | 1.0996 | 1.0996 |  | 1.0029 | 1.0396 | 1.0593 | 1.0625 | 1.0823 | 1.0938 |  | 0.9977 | 1.0123 | 1.0203 | 1.0570 | 1.0653 | 1.0881 |
| 2.4 | 1.0286 | 1.0847 | 1.1139 | 1.0847 | 1.1139 | 1.1139 |  | 1.0234 | 1.0582 | 1.0768 | 1.0794 | 1.0980 | 1.1086 |  | 1.0185 | 1.0327 | 1.0404 | 1.0742 | 1.0822 | 1.1033 |
| 2.2 | 1.0492 | 1.1014 | 1.1286 | 1.1014 | 1.1286 | 1.1286 |  | 1.0444 | 1.0772 | 1.0946 | 1.0965 | 1.1140 | 1.1237 |  | 1.0398 | 1.0535 | 1.0609 | 1.0918 | 1.0994 | 1.1188 |
| 2 | 1.0702 | 1.1185 | 1.1435 | 1.1185 | 1.1435 | 1.1435 |  | 1.0658 | 1.0965 | 1.1127 | 1.1141 | 1.1303 | 1.1391 |  | 1.0616 | 1.0747 | 1.0817 | 1.1097 | 1.1169 | 1.1346 |
| 1.8 | 1.0917 | 1.1359 | 1.1587 | 1.1359 | 1.1587 | 1.1587 |  | 1.0878 | 1.1162 | 1.1311 | 1.1320 | 1.1469 | 1.1548 |  | 1.0839 | 1.0963 | 1.1029 | 1.1280 | 1.1348 | 1.1507 |
| 1.6 | 1.1138 | 1.1537 | 1.1743 | 1.1537 | 1.1743 | 1.1743 |  | 1.1103 | 1.1362 | 1.1498 | 1.1502 | 1.1638 | 1.1708 |  | 1.1068 | 1.1184 | 1.1245 | 1.1466 | 1.1529 | 1.1672 |
| 1.4 | 1.1363 | 1.1719 | 1.1902 | 1.1719 | 1.1902 | 1.1902 |  | 1.1333 | 1.1566 | 1.1688 | 1.1689 | 1.1810 | 1.1871 |  | 1.1302 | 1.1408 | 1.1465 | 1.1657 | 1.1714 | 1.1839 |
| 1.2 | 1.1594 | 1.1905 | 1.2064 | 1.1905 | 1.2064 | 1.2064 |  | 1.1568 | 1.1774 | 1.1881 | 1.1879 | 1.1985 | 1.2037 |  | 1.1541 | 1.1637 | 1.1688 | 1.1851 | 1.1902 | 1.2010 |
| 1 | 1.1831 | 1.2094 | 1.2229 | 1.2094 | 1.2229 | 1.2229 |  | 1.1809 | 1.1986 | 1.2077 | 1.2073 | 1.2164 | 1.2207 |  | 1.1786 | 1.1871 | 1.1915 | 1.2050 | 1.2094 | 1.2184 |

| $\lambda$ | Time-to-immunity = 21 days | | | | | | | | | | | | | | | | | | | |
| --- | --- | --- | --- | --- | --- | --- | --- | --- | --- | --- | --- | --- | --- | --- | --- | --- | --- | --- | --- | --- |
|  | Mutation Time = Day 10 | | | | | |  | Mutation Time = Day 30 | | | | | |  | Mutation Time = Day 50 | | | | | |
|  | $H_{i}H_{f}$ | $H_{i}M_{f}$ | $H_{i}L_{f}$ | $M_{i}M_{f}$ | $M_{i}L_{f}$ | $L_{i}L_{f}$ |  | $H_{i}H_{f}$ | $H_{i}M_{f}$ | $H_{i}L_{f}$ | $M_{i}M_{f}$ | $M_{i}L_{f}$ | $L_{i}L_{f}$ |  | $H_{i}H_{f}$ | $H_{i}M_{f}$ | $H_{i}L_{f}$ | $M_{i}M_{f}$ | $M_{i}L_{f}$ | $L_{i}L_{f}$ |
| 3 | 1.0237 | 1.0785 | 1.1076 | 1.0785 | 1.1076 | 1.1076 |  | 1.0202 | 1.0605 | 1.0822 | 1.0749 | 1.0967 | 1.1040 |  | 1.0142 | 1.0296 | 1.0379 | 1.0686 | 1.0774 | 1.0975 |
| 2.8 | 1.0393 | 1.0914 | 1.1190 | 1.0914 | 1.1190 | 1.1190 |  | 1.0360 | 1.0746 | 1.0953 | 1.0880 | 1.1088 | 1.1156 |  | 1.0304 | 1.0454 | 1.0535 | 1.0821 | 1.0907 | 1.1095 |
| 2.6 | 1.0553 | 1.1046 | 1.1306 | 1.1046 | 1.1306 | 1.1306 |  | 1.0522 | 1.0890 | 1.1086 | 1.1015 | 1.1212 | 1.1275 |  | 1.0469 | 1.0615 | 1.0695 | 1.0959 | 1.1042 | 1.1218 |
| 2.4 | 1.0718 | 1.1181 | 1.1425 | 1.1181 | 1.1425 | 1.1425 |  | 1.0689 | 1.1037 | 1.1222 | 1.1153 | 1.1338 | 1.1396 |  | 1.0639 | 1.0781 | 1.0857 | 1.1101 | 1.1180 | 1.1343 |
| 2.2 | 1.0887 | 1.1320 | 1.1547 | 1.1320 | 1.1547 | 1.1547 |  | 1.0860 | 1.1188 | 1.1361 | 1.1294 | 1.1468 | 1.1521 |  | 1.0814 | 1.0950 | 1.1024 | 1.1246 | 1.1322 | 1.1472 |
| 2 | 1.1060 | 1.1463 | 1.1672 | 1.1463 | 1.1672 | 1.1672 |  | 1.1037 | 1.1342 | 1.1503 | 1.1438 | 1.1600 | 1.1648 |  | 1.0994 | 1.1124 | 1.1194 | 1.1394 | 1.1466 | 1.1603 |
| 1.8 | 1.1239 | 1.1608 | 1.1800 | 1.1608 | 1.1800 | 1.1800 |  | 1.1218 | 1.1500 | 1.1648 | 1.1587 | 1.1735 | 1.1779 |  | 1.1179 | 1.1302 | 1.1367 | 1.1547 | 1.1614 | 1.1738 |
| 1.6 | 1.1423 | 1.1758 | 1.1931 | 1.1758 | 1.1931 | 1.1931 |  | 1.1404 | 1.1662 | 1.1797 | 1.1739 | 1.1874 | 1.1912 |  | 1.1369 | 1.1484 | 1.1544 | 1.1703 | 1.1765 | 1.1876 |
| 1.4 | 1.1612 | 1.1912 | 1.2066 | 1.1912 | 1.2066 | 1.2066 |  | 1.1595 | 1.1827 | 1.1948 | 1.1895 | 1.2015 | 1.2049 |  | 1.1564 | 1.1670 | 1.1726 | 1.1863 | 1.1920 | 1.2017 |
| 1.2 | 1.1807 | 1.2069 | 1.2204 | 1.2069 | 1.2204 | 1.2204 |  | 1.1792 | 1.1997 | 1.2103 | 1.2055 | 1.2161 | 1.2189 |  | 1.1765 | 1.1860 | 1.1910 | 1.2027 | 1.2078 | 1.2162 |
| 1 | 1.2007 | 1.2230 | 1.2345 | 1.2230 | 1.2345 | 1.2345 |  | 1.1995 | 1.2171 | 1.2261 | 1.2218 | 1.2309 | 1.2333 |  | 1.1972 | 1.2056 | 1.2099 | 1.2195 | 1.2240 | 1.2310 |

Table S4: Mortality rate under different times-to-immunity, mutation times, and capacity multipliers for each vaccine type

- 1. **Minimum required capacity multiplier of each vaccine type under different mutation times to achieve a lower IAR than vaccine-**$\boldsymbol{H}_{\boldsymbol{i}}\boldsymbol{H}_{\boldsymbol{f}}$

We simulated with a step size of 0.01 to compute the minimum capacity multiplier for which each vaccine type achieves a lower IAR than vaccine-$H_{i}H_{f}$ when its capacity multiplier is 1.0, 1.2, 1.4, 1.6, and 2.0. Table S5 shows that as the variants emerge later (i.e., higher mutation time), the minimum capacity multiplier for all vaccine types is lower. When the mutation time is early, the minimum capacity multiplier is smaller for vaccine-$M_{i}M_{f}$ than vaccine-$H_{i}L_{f}$ as observed in Table S1, where vaccine-$M_{i}M_{f}$ achieves lower IAR than vaccine-$H_{i}L_{f}$ . In addition, as the capacity multiplier of vaccine-$H_{i}H_{f}$ becomes larger, the difference of the minimum capacity multiplier between when the mutation time is early and late also becomes larger. For example, when the capacity multiplier of vaccine-$H_{i}H_{f}$ is 1.0, the minimum capacity multiplier of vaccine-$H_{i}L_{f}$ is 1.38 and 1.09 when the mutation time is Day 10 and Day 50, respectively (difference= 0.29). On the other hand, when the capacity multiplier of vaccine-$H_{i}H_{f}$ is 2.0, the minimum capacity multiplier of vaccine-$H_{i}L_{f}$ is 2.74 and 2.15 when the mutation time is Day 10 and Day 50, respectively (difference= 0.59).

| Mutation | Capacity multiplier = 1.0 | | | | |  | Capacity multiplier = 1.2 | | | | |
| --- | --- | --- | --- | --- | --- | --- | --- | --- | --- | --- | --- |
| Time | $H_{i}M_{f}$ | $H_{i}L_{f}$ | $M_{i}M_{f}$ | $M_{i}L_{f}$ | $L_{i}L_{f}$ |  | $H_{i}M_{f}$ | $H_{i}L_{f}$ | $M_{i}M_{f}$ | $M_{i}L_{f}$ | $L_{i}L_{f}$ |
| 5 | 1.25 | 1.43 | 1.28 | 1.47 | 1.49 |  | 1.5 | 1.72 | 1.54 | 1.77 | 1.79 |
| 10 | 1.22 | 1.38 | 1.28 | 1.45 | 1.49 |  | 1.47 | 1.65 | 1.54 | 1.74 | 1.79 |
| 15 | 1.2 | 1.33 | 1.28 | 1.43 | 1.49 |  | 1.43 | 1.59 | 1.54 | 1.71 | 1.78 |
| 20 | 1.17 | 1.28 | 1.28 | 1.41 | 1.48 |  | 1.4 | 1.53 | 1.53 | 1.69 | 1.78 |
| 25 | 1.15 | 1.24 | 1.28 | 1.39 | 1.48 |  | 1.38 | 1.48 | 1.53 | 1.67 | 1.78 |
| 30 | 1.13 | 1.2 | 1.28 | 1.37 | 1.48 |  | 1.35 | 1.44 | 1.53 | 1.64 | 1.77 |
| 35 | 1.11 | 1.17 | 1.27 | 1.36 | 1.47 |  | 1.33 | 1.4 | 1.53 | 1.62 | 1.77 |
| 40 | 1.09 | 1.14 | 1.27 | 1.34 | 1.47 |  | 1.3 | 1.36 | 1.53 | 1.61 | 1.77 |
| 45 | 1.07 | 1.11 | 1.27 | 1.33 | 1.47 |  | 1.29 | 1.33 | 1.53 | 1.59 | 1.76 |
| 50 | 1.06 | 1.09 | 1.27 | 1.31 | 1.47 |  | 1.27 | 1.31 | 1.53 | 1.58 | 1.76 |
| 55 | 1.05 | 1.07 | 1.27 | 1.3 | 1.47 |  | 1.25 | 1.28 | 1.52 | 1.56 | 1.76 |
| 60 | 1.04 | 1.06 | 1.27 | 1.3 | 1.46 |  | 1.24 | 1.26 | 1.52 | 1.55 | 1.76 |

| Mutation | Capacity multiplier = 1.4 | | | | |  | Capacity multiplier = 1.6 | | | | |
| --- | --- | --- | --- | --- | --- | --- | --- | --- | --- | --- | --- |
| Time | $H_{i}M_{f}$ | $H_{i}L_{f}$ | $M_{i}M_{f}$ | $M_{i}L_{f}$ | $L_{i}L_{f}$ |  | $H_{i}M_{f}$ | $H_{i}L_{f}$ | $M_{i}M_{f}$ | $M_{i}L_{f}$ | $L_{i}L_{f}$ |
| 5 | 1.75 | 2.01 | 1.8 | 2.06 | 2.09 |  | 2 | 2.29 | 2.06 | 2.36 | 2.4 |
| 10 | 1.71 | 1.92 | 1.8 | 2.03 | 2.09 |  | 1.95 | 2.2 | 2.05 | 2.32 | 2.39 |
| 15 | 1.67 | 1.85 | 1.79 | 2 | 2.09 |  | 1.91 | 2.11 | 2.05 | 2.29 | 2.39 |
| 20 | 1.64 | 1.78 | 1.79 | 1.97 | 2.08 |  | 1.87 | 2.03 | 2.05 | 2.25 | 2.38 |
| 25 | 1.6 | 1.73 | 1.79 | 1.94 | 2.08 |  | 1.83 | 1.97 | 2.05 | 2.22 | 2.38 |
| 30 | 1.57 | 1.67 | 1.79 | 1.92 | 2.07 |  | 1.79 | 1.91 | 2.04 | 2.19 | 2.37 |
| 35 | 1.54 | 1.63 | 1.78 | 1.89 | 2.07 |  | 1.76 | 1.85 | 2.04 | 2.16 | 2.37 |
| 40 | 1.52 | 1.58 | 1.78 | 1.87 | 2.06 |  | 1.73 | 1.8 | 2.04 | 2.14 | 2.36 |
| 45 | 1.5 | 1.55 | 1.78 | 1.85 | 2.06 |  | 1.71 | 1.76 | 2.04 | 2.12 | 2.36 |
| 50 | 1.48 | 1.52 | 1.78 | 1.84 | 2.06 |  | 1.68 | 1.73 | 2.03 | 2.1 | 2.35 |
| 55 | 1.46 | 1.49 | 1.78 | 1.82 | 2.05 |  | 1.67 | 1.7 | 2.03 | 2.08 | 2.35 |
| 60 | 1.45 | 1.47 | 1.78 | 1.81 | 2.05 |  | 1.65 | 1.68 | 2.03 | 2.07 | 2.35 |

| Mutation | Capacity multiplier = 1.8 | | | | |  | Capacity multiplier = 2.0 | | | | |
| --- | --- | --- | --- | --- | --- | --- | --- | --- | --- | --- | --- |
| Time | $H_{i}M_{f}$ | $H_{i}L_{f}$ | $M_{i}M_{f}$ | $M_{i}L_{f}$ | $L_{i}L_{f}$ |  | $H_{i}M_{f}$ | $H_{i}L_{f}$ | $M_{i}M_{f}$ | $M_{i}L_{f}$ | $L_{i}L_{f}$ |
| 5 | 2.25 | 2.58 | 2.31 | 2.66 | 2.7 |  | 2.51 | 2.87 | 2.57 | 2.96 | 3.01 |
| 10 | 2.2 | 2.47 | 2.31 | 2.62 | 2.7 |  | 2.44 | 2.74 | 2.57 | 2.91 | 3 |
| 15 | 2.14 | 2.37 | 2.31 | 2.57 | 2.69 |  | 2.38 | 2.63 | 2.57 | 2.86 | 2.99 |
| 20 | 2.1 | 2.28 | 2.31 | 2.53 | 2.68 |  | 2.32 | 2.53 | 2.56 | 2.82 | 2.99 |
| 25 | 2.05 | 2.21 | 2.3 | 2.5 | 2.68 |  | 2.28 | 2.44 | 2.56 | 2.77 | 2.98 |
| 30 | 2.01 | 2.14 | 2.3 | 2.46 | 2.67 |  | 2.23 | 2.37 | 2.56 | 2.74 | 2.97 |
| 35 | 1.98 | 2.08 | 2.3 | 2.43 | 2.67 |  | 2.19 | 2.3 | 2.55 | 2.7 | 2.97 |
| 40 | 1.94 | 2.02 | 2.29 | 2.4 | 2.66 |  | 2.16 | 2.24 | 2.55 | 2.67 | 2.96 |
| 45 | 1.92 | 1.98 | 2.29 | 2.38 | 2.66 |  | 2.12 | 2.19 | 2.55 | 2.64 | 2.96 |
| 50 | 1.89 | 1.94 | 2.29 | 2.36 | 2.65 |  | 2.1 | 2.15 | 2.55 | 2.62 | 2.95 |
| 55 | 1.87 | 1.91 | 2.29 | 2.34 | 2.65 |  | 2.08 | 2.11 | 2.54 | 2.6 | 2.95 |
| 60 | 1.85 | 1.88 | 2.29 | 2.33 | 2.65 |  | 2.06 | 2.09 | 2.54 | 2.58 | 2.95 |

Table S5: Minimum capacity multiplier under different mutation times to achieve a lower IAR than vaccine-$H_{i}H_{f}$

1. **Extended SIR-D Model**
   1. **Compartmental Model**

We extended the main model to capture the dynamics of COVID-19 transmission and the impacts of emergence of variants more comprehensively and show the robustness of our results. The list below summarizes the compartments we included in the extended model:

1. Susceptible
   1. $S$: Susceptible
   2. $S^{V_{i}}:$ (Vaccinated-susceptible) Population who has received vaccine before the variants but remains susceptible or becomes susceptible after the variants
   3. $S^{V_{f}}$: (Vaccinated-susceptible after variants) Population who has received vaccine after the variants but remains susceptible
2. Infected
   1. $I_{S}$: Symptomatic infected (unvaccinated)
   2. $I_{A}$: Asymptomatic infected (unvaccinated)
   3. $I_{S}^{V}$: Vaccinated but later got symptomatic-infected
   4. $I_{A}^{V}$: Vaccinated but later got asymptomatic-infected
3. Vaccinated (Immunized)
   1. $V_{i}:$ Vaccinated with immunity *before* the emergence of variants
   2. $V_{f}$: Vaccinated with immunity *after* the emergence of variants
4. Recovered
   1. $R$: Recovered
5. Deceased
   1. $D:$ Deceased

Figure S2 depicts the transition diagrams of the extended model before and after the mutation time, using the epidemiological and vaccine parameters summarized in Table S6. In this extended model, we first changed the notation for the *Vaccinated with immunity* compartment from $V$ to $V_{i}$ and the *Vaccinated-susceptible* compartment from $S^{V}$ to $S^{V_{i}}$. We then made two major modifications:

First, we added two additional infected compartments ($I_{A}^{V}$ and $I_{S}^{V}$), to which the vaccinated but susceptible individuals move when they get infected, to differentiate their death rates $\mu^{V}$ from the unvaccinated individuals’ $\mu$. This modification reflects the reduction in mortality risk observed in vaccinated populations compared to unvaccinated populations.^2,3^ Regardless of the vaccination status, we set the individuals to have the same recovery rate ($\gamma_{S}$ for symptomatic patients or $\gamma_{A}$ for asymptomatic patients), supported by the isolation policy by the Centers for Disease Control and Prevention (CDC).^4^

Second, we added additional vaccination and susceptible compartments to capture the individuals’ immunity against the virus *after* the mutation time. Before the mutation time, the extended model is similar to the main model (simply with the first modification stated above). After the mutation time, however, the extended model has two new compartments: *Vaccinated-with immunity after variants* ($V_{f}$) and *Vaccinated-susceptible after variants* ($S^{V_{f}}$).

While individuals who are vaccinated are fully protected throughout the *entire* time horizon in the main model, they may lose immunity after the mutation time in the extended model. Specifically, the mutation time influences the transitions of the susceptible population and the vaccinated population in the extended model as follows:

1. (*Before the mutation time*) individuals who receive vaccines enter the *Vaccinated with immunity* compartment ($V_{i}$) if they build immunity based on the efficacy of the vaccine (i.e., $S\to V_{i}$ if the vaccine is effective, and ${S\to S}^{V_{i}}$, otherwise).
2. (*After the mutation time*) individuals in compartment $V_{i}$ become susceptible again as the previously obtained immunity may not protect them from the variants (i.e., $V_{i}\to S^{V_{i}}$)
3. (*After the mutation time*) individuals who are newly vaccinated or re-vaccinated (e.g., booster shot) enter the *Vaccinated with immunity after variants* compartment ($V_{f}$) if the vaccine is effective and the *Vaccinated-susceptible after variants* ($S^{V_{f}}$) otherwise (i.e., $S$ and $S^{V_{i}}\to$ $V_{f}$ if effective, and $S$ and $S^{V_{i}}$ $\to$ $S^{V_{f}}$, otherwise). The vaccine is administered to the individuals in compartment $S$ and compartment $S^{V_{i}}$ on a first-come-first-served basis.

| 1. Before mutation time |
| --- |
| 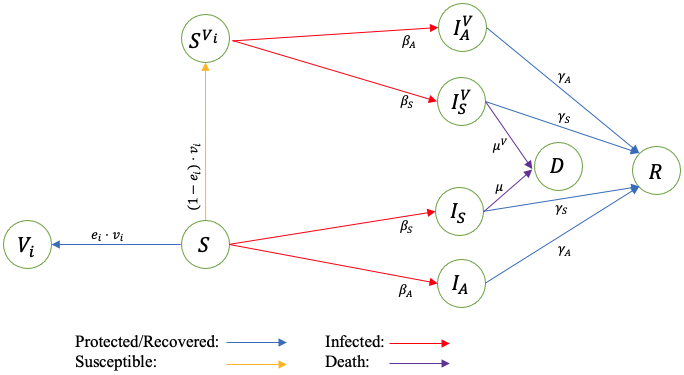 |
|  |
| 1. After mutation time |
| 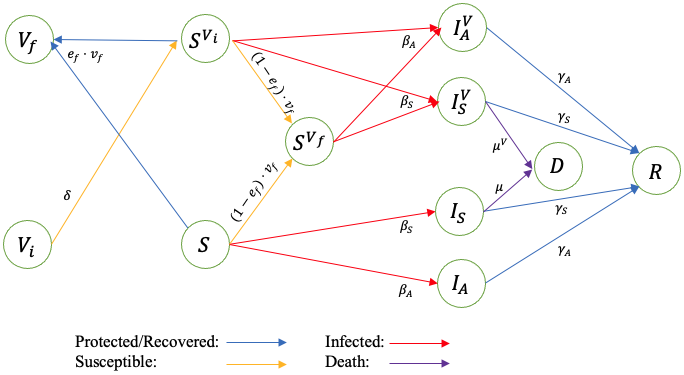 |

Figure S2: Transition diagrams of the extended SIR-D model, in which each move is dependent on various parameters, including vaccine efficacy before and after virus mutation. $\beta_{S}, \beta_{A}:$ symptomatic and asymptomatic transmission rates, respectively; $\gamma_{S},\gamma_{A}$: symptomatic and asymptomatic recovery rates, respectively; $\mu^{V},\mu$: decease rate of a symptomatic patient who is vaccinated and unvaccinated, respectively; $e_{i},e_{f}$: efficacy of the vaccine before and after mutation time, respectively; $v_{i}, v_{f}$: daily vaccinated individuals before and after mutation time, respectively.

| **Epidemiological Parameters** | Value | Description |
| --- | --- | --- |
| $\beta_{S}$ | $0.219328$ | Rate of disease transmission from symptomatic-infected individuals |
| $\beta_{A}$ | $0.164496$ | Rate of disease transmission from asymptomatic-infected individuals |
| $\gamma_{S}$ | $1/14$ | Rate of recovery for symptomatic-infected individuals |
| $\gamma_{A}$ | $1/8$ | Rate of recovery for asymptomatic-infected individuals |
| $\mu$ | $0.0015$ | Rate of death among symptomatic-infected unvaccinated individuals |
| $\mu^{V}$ | $0.0015/12.7$ | Rate of death among symptomatic-infected vaccinated individuals |
| $p_{S}$ | 70% | Percentage of symptomatic infection |
|  |  |  |
| **Vaccine Parameters** | Value | Description |
| $K$ | 500,000 | Base vaccine capacity |
| $\lambda$ | 1.0 to 3.0 with increments of 0.2 | Capacity multiplier |
| $e_{i}$ | Initial Efficacy in Table 1 | Efficacy before the emergence of variants |
| $e_{f}$ | Final Efficacy in Table 1 | Efficacy after the emergence of variants |
| $v_{i}$ | 0 or $min(1,\frac{\lambda\cdot K}{S\cdot N})$ | Daily vaccinated individuals before the mutation time ($N$= population size; 330 million)^[[1]](#footnote-1)^ |
| $v_{f}$ | 0 or $min(1,\frac{\lambda\cdot K}{(S+S^{V_{i}})\cdot N})$ | Daily vaccinated individuals at and after the mutation time^[[2]](#footnote-2)^ |
| $\delta$ | $0$ or $1/7$ | Rate of losing immunity due to variants^[[3]](#footnote-3)^ |

Table S6: Epidemiological and vaccine parameters used in the extended model

The non-linear system of ordinary differential equations (ODEs) that govern the movements among the compartments in the extended model is as follows:

$$\frac{dS}{dt}=-(v_{i}+v_{f})\cdot S-\beta_{S}\cdot\left( 1-v_{i}-v_{f} \right)\cdot S\cdot\left( I_{S}+I_{S}^{V} \right)-\beta_{A}\cdot\left( 1-v_{i}-v_{f} \right)\cdot S\cdot(I_{A}+I_{A}^{V})$$

$$\frac{dS^{V_{i}}}{dt}=\left( 1-e_{i} \right)\cdot v_{i}\cdot S+\delta V_{i}-\beta_{S}S^{V_{i}}\left( I_{S}+I_{S}^{V} \right)-\beta_{A}S^{V_{i}}\left( I_{A}+I_{A}^{V} \right)-v_{f}\cdot S^{V_{i}}$$

$$\frac{dS^{V_{f}}}{dt}=\left( 1-e_{f} \right)\cdot v_{f}\cdot S+\left( 1-e_{f} \right)\cdot v_{f}\cdot S^{V_{i}}-\beta_{S}S^{V_{f}}\left( I_{S}+I_{S}^{V} \right)-\beta_{A}S^{V_{f}}(I_{A}+I_{A}^{V})$$

$$\frac{dV_{i}}{dt}=e_{i}\cdot v_{i}\cdot S-\delta V_{i}$$

$$\frac{dV_{f}}{dt}=e_{f}\cdot v_{f}\cdot S+e_{f}\cdot v_{f}\cdot S^{V_{i}}$$

$$\frac{dI_{S}}{dt}=p_{S}\cdot\left[ \beta_{S}\cdot\left( 1-v_{i}-v_{f} \right)\cdot(I_{S}+I_{S}^{V})+\beta_{A}\cdot\left( 1-v_{i}-v_{f} \right)\cdot{(I}_{A}+I_{A}^{V}) \right]\cdot S-\gamma_{S}I_{S}-\mu I_{S}$$

$$\frac{dI_{A}}{dt}={(1-p}_{S})\cdot\left[ \beta_{S}\cdot\left( 1-v_{i}-v_{f} \right)\cdot(I_{S}+I_{S}^{V})+\beta_{A}\cdot\left( 1-v_{i}-v_{f} \right){\cdot(I}_{A}+I_{A}^{V}) \right]\cdot S-\gamma_{A}I_{A}$$

$$\frac{dI_{S}^{V}}{dt}= p_{S}\cdot\left( \beta_{S}\cdot S^{V_{i}}\cdot\left( I_{S}+I_{S}^{V} \right)+\beta_{A}\cdot S^{V_{i}}\cdot{(I}_{A}+I_{A}^{V})+\beta_{S}\cdot S^{V_{f}}\cdot\left( I_{S}+I_{S}^{V} \right)+\beta_{A}\cdot S^{V_{f}}\cdot{(I}_{A}+I_{A}^{V}) \right)-\gamma_{S}I_{S}^{V}-\mu^{V}I_{S}^{V}$$

$$\frac{dI_{A}^{V}}{dt}=(1-p_{S})\cdot\left( \beta_{S}\cdot S^{V_{i}}\cdot\left( I_{S}+I_{S}^{V} \right)+\beta_{A}\cdot S^{V_{i}}\cdot{(I}_{A}+I_{A}^{V})+\beta_{S}\cdot S^{V_{f}}\cdot\left( I_{S}+I_{S}^{V} \right)+\beta_{A}\cdot S^{V_{f}}\cdot{(I}_{A}+I_{A}^{V}) \right)-\gamma_{A}I_{A}^{V}$$

$$\frac{dR}{dt}=\gamma_{S}I_{S}+\gamma_{A}I_{A}+\gamma_{S}I_{S}^{V}+\gamma_{A}I_{A}^{V}$$

$$\frac{dD}{dt}=\mu I_{S}+\mu^{V}I_{S}^{V}$$

- 1. **Simulation Results with the Extended SIR-D Model**

Table S7 and Table S8 show the full results of the simulation with the extended model under different vaccine types and capacity multipliers ($\lambda$) for a given mutation time when the main outcome of interest is the infection attack rate (IAR) and the mortality rate, respectively.

| Capacity Multiplier $(\lambda)$ | Mutation Time = Day 5 | | | | | |  | Mutation Time = Day 10 | | | | | |
| --- | --- | --- | --- | --- | --- | --- | --- | --- | --- | --- | --- | --- | --- |
|  | $H_{i}H_{f}$ | $H_{i}M_{f}$ | $H_{i}L_{f}$ | $M_{i}M_{f}$ | $M_{i}L_{f}$ | $L_{i}L_{f}$ |  | $H_{i}H_{f}$ | $H_{i}M_{f}$ | $H_{i}L_{f}$ | $M_{i}M_{f}$ | $M_{i}L_{f}$ | $L_{i}L_{f}$ |
| 3 | 58.09 | 59.87 | 60.70 | 59.91 | 60.74 | 60.76 |  | 60.76 | 62.25 | 62.94 | 62.37 | 63.06 | 63.12 |
| 2.8 | 59.88 | 61.55 | 62.33 | 61.59 | 62.37 | 62.39 |  | 62.39 | 63.79 | 64.45 | 63.90 | 64.55 | 64.60 |
| 2.6 | 61.71 | 63.27 | 64.00 | 63.31 | 64.04 | 64.05 |  | 64.06 | 65.37 | 65.99 | 65.47 | 66.08 | 66.12 |
| 2.4 | 63.57 | 65.03 | 65.71 | 65.06 | 65.74 | 65.75 |  | 65.76 | 66.98 | 67.56 | 67.06 | 67.64 | 67.67 |
| 2.2 | 65.48 | 66.82 | 67.45 | 66.85 | 67.47 | 67.49 |  | 67.49 | 68.63 | 69.16 | 68.69 | 69.23 | 69.26 |
| 2 | 67.43 | 68.65 | 69.23 | 68.67 | 69.25 | 69.26 |  | 69.26 | 70.30 | 70.79 | 70.36 | 70.85 | 70.88 |
| 1.8 | 69.41 | 70.52 | 71.04 | 70.53 | 71.06 | 71.06 |  | 71.06 | 72.01 | 72.46 | 72.06 | 72.50 | 72.53 |
| 1.6 | 71.43 | 72.42 | 72.89 | 72.43 | 72.90 | 72.91 |  | 72.90 | 73.75 | 74.16 | 73.79 | 74.19 | 74.21 |
| 1.4 | 73.49 | 74.36 | 74.77 | 74.37 | 74.78 | 74.79 |  | 74.78 | 75.53 | 75.89 | 75.56 | 75.92 | 75.93 |
| 1.2 | 75.58 | 76.33 | 76.69 | 76.34 | 76.70 | 76.71 |  | 76.69 | 77.34 | 77.66 | 77.36 | 77.68 | 77.69 |
| 1 | 77.71 | 78.35 | 78.65 | 78.35 | 78.66 | 78.66 |  | 78.63 | 79.19 | 79.46 | 79.20 | 79.47 | 79.48 |

| Capacity Multiplier $(\lambda)$ | Mutation Time = Day 15 | | | | | |  | Mutation Time = Day 20 | | | | | |
| --- | --- | --- | --- | --- | --- | --- | --- | --- | --- | --- | --- | --- | --- |
|  | $H_{i}H_{f}$ | $H_{i}M_{f}$ | $H_{i}L_{f}$ | $M_{i}M_{f}$ | $M_{i}L_{f}$ | $L_{i}L_{f}$ |  | $H_{i}H_{f}$ | $H_{i}M_{f}$ | $H_{i}L_{f}$ | $M_{i}M_{f}$ | $M_{i}L_{f}$ | $L_{i}L_{f}$ |
| 3 | 63.15 | 64.39 | 64.98 | 64.62 | 65.20 | 65.31 |  | 65.23 | 66.28 | 66.78 | 66.64 | 67.13 | 67.30 |
| 2.8 | 64.65 | 65.83 | 66.38 | 66.03 | 66.57 | 66.67 |  | 66.63 | 67.62 | 68.09 | 67.94 | 68.40 | 68.56 |
| 2.6 | 66.18 | 67.28 | 67.81 | 67.46 | 67.98 | 68.06 |  | 68.05 | 68.98 | 69.43 | 69.27 | 69.70 | 69.84 |
| 2.4 | 67.74 | 68.77 | 69.26 | 68.92 | 69.41 | 69.48 |  | 69.50 | 70.37 | 70.78 | 70.62 | 71.02 | 71.14 |
| 2.2 | 69.33 | 70.28 | 70.74 | 70.41 | 70.86 | 70.93 |  | 70.96 | 71.77 | 72.16 | 71.99 | 72.37 | 72.47 |
| 2 | 70.94 | 71.83 | 72.25 | 71.94 | 72.35 | 72.40 |  | 72.46 | 73.20 | 73.56 | 73.39 | 73.74 | 73.83 |
| 1.8 | 72.59 | 73.40 | 73.78 | 73.49 | 73.87 | 73.91 |  | 73.97 | 74.66 | 74.99 | 74.81 | 75.13 | 75.21 |
| 1.6 | 74.27 | 75.00 | 75.35 | 75.07 | 75.42 | 75.45 |  | 75.52 | 76.14 | 76.44 | 76.26 | 76.56 | 76.62 |
| 1.4 | 75.98 | 76.63 | 76.94 | 76.69 | 77.00 | 77.03 |  | 77.09 | 77.64 | 77.91 | 77.74 | 78.01 | 78.06 |
| 1.2 | 77.73 | 78.29 | 78.57 | 78.34 | 78.61 | 78.63 |  | 78.68 | 79.17 | 79.41 | 79.25 | 79.49 | 79.52 |
| 1 | 79.51 | 79.99 | 80.22 | 80.02 | 80.26 | 80.27 |  | 80.31 | 80.73 | 80.94 | 80.79 | 80.99 | 81.02 |

| Capacity Multiplier $(\lambda)$ | Mutation Time = Day 25 | | | | | |  | Mutation Time = Day 30 | | | | | |
| --- | --- | --- | --- | --- | --- | --- | --- | --- | --- | --- | --- | --- | --- |
|  | $H_{i}H_{f}$ | $H_{i}M_{f}$ | $H_{i}L_{f}$ | $M_{i}M_{f}$ | $M_{i}L_{f}$ | $L_{i}L_{f}$ |  | $H_{i}H_{f}$ | $H_{i}M_{f}$ | $H_{i}L_{f}$ | $M_{i}M_{f}$ | $M_{i}L_{f}$ | $L_{i}L_{f}$ |
| 3 | 66.97 | 67.86 | 68.28 | 68.40 | 68.81 | 69.07 |  | 68.33 | 69.09 | 69.46 | 69.85 | 70.20 | 70.57 |
| 2.8 | 68.30 | 69.13 | 69.54 | 69.62 | 70.00 | 70.24 |  | 69.60 | 70.32 | 70.67 | 71.01 | 71.33 | 71.67 |
| 2.6 | 69.64 | 70.42 | 70.80 | 70.85 | 71.22 | 71.43 |  | 70.89 | 71.57 | 71.89 | 72.18 | 72.49 | 72.79 |
| 2.4 | 70.99 | 71.73 | 72.09 | 72.11 | 72.45 | 72.64 |  | 72.19 | 72.82 | 73.13 | 73.36 | 73.65 | 73.92 |
| 2.2 | 72.37 | 73.05 | 73.39 | 73.38 | 73.70 | 73.86 |  | 73.50 | 74.09 | 74.37 | 74.56 | 74.83 | 75.06 |
| 2 | 73.76 | 74.40 | 74.70 | 74.68 | 74.97 | 75.11 |  | 74.82 | 75.36 | 75.63 | 75.77 | 76.03 | 76.23 |
| 1.8 | 75.17 | 75.76 | 76.04 | 75.99 | 76.27 | 76.38 |  | 76.16 | 76.66 | 76.90 | 77.00 | 77.24 | 77.41 |
| 1.6 | 76.61 | 77.14 | 77.39 | 77.33 | 77.58 | 77.68 |  | 77.51 | 77.96 | 78.18 | 78.25 | 78.47 | 78.61 |
| 1.4 | 78.06 | 78.53 | 78.77 | 78.69 | 78.92 | 79.00 |  | 78.87 | 79.28 | 79.48 | 79.52 | 79.71 | 79.83 |
| 1.2 | 79.54 | 79.96 | 80.16 | 80.08 | 80.28 | 80.35 |  | 80.25 | 80.61 | 80.79 | 80.80 | 80.98 | 81.07 |
| 1 | 81.04 | 81.40 | 81.58 | 81.49 | 81.67 | 81.72 |  | 81.65 | 81.96 | 82.12 | 82.11 | 82.26 | 82.33 |

| Capacity Multiplier $(\lambda)$ | Mutation Time = Day 35 | | | | | |  | Mutation Time = Day 40 | | | | | |
| --- | --- | --- | --- | --- | --- | --- | --- | --- | --- | --- | --- | --- | --- |
|  | $H_{i}H_{f}$ | $H_{i}M_{f}$ | $H_{i}L_{f}$ | $M_{i}M_{f}$ | $M_{i}L_{f}$ | $L_{i}L_{f}$ |  | $H_{i}H_{f}$ | $H_{i}M_{f}$ | $H_{i}L_{f}$ | $M_{i}M_{f}$ | $M_{i}L_{f}$ | $L_{i}L_{f}$ |
| 3 | 69.25 | 69.92 | 70.25 | 70.95 | 71.24 | 71.76 |  | 69.72 | 70.32 | 70.62 | 71.65 | 71.91 | 72.58 |
| 2.8 | 70.51 | 71.14 | 71.45 | 72.07 | 72.35 | 72.81 |  | 70.98 | 71.55 | 71.82 | 72.76 | 73.00 | 73.61 |
| 2.6 | 71.77 | 72.36 | 72.65 | 73.19 | 73.46 | 73.87 |  | 72.25 | 72.77 | 73.03 | 73.87 | 74.09 | 74.65 |
| 2.4 | 73.04 | 73.59 | 73.86 | 74.33 | 74.58 | 74.95 |  | 73.51 | 74.00 | 74.24 | 74.98 | 75.19 | 75.69 |
| 2.2 | 74.31 | 74.82 | 75.07 | 75.48 | 75.71 | 76.03 |  | 74.77 | 75.23 | 75.45 | 76.10 | 76.30 | 76.74 |
| 2 | 75.59 | 76.07 | 76.30 | 76.63 | 76.85 | 77.13 |  | 76.04 | 76.46 | 76.66 | 77.22 | 77.41 | 77.80 |
| 1.8 | 76.88 | 77.31 | 77.53 | 77.80 | 78.00 | 78.25 |  | 77.31 | 77.69 | 77.88 | 78.36 | 78.53 | 78.86 |
| 1.6 | 78.18 | 78.57 | 78.76 | 78.98 | 79.17 | 79.38 |  | 78.58 | 78.93 | 79.10 | 79.50 | 79.65 | 79.94 |
| 1.4 | 79.48 | 79.83 | 80.01 | 80.18 | 80.35 | 80.52 |  | 79.85 | 80.16 | 80.32 | 80.65 | 80.79 | 81.03 |
| 1.2 | 80.79 | 81.11 | 81.26 | 81.39 | 81.54 | 81.68 |  | 81.13 | 81.41 | 81.54 | 81.80 | 81.93 | 82.13 |
| 1 | 82.12 | 82.39 | 82.52 | 82.61 | 82.74 | 82.85 |  | 82.42 | 82.65 | 82.77 | 82.97 | 83.08 | 83.24 |

| Capacity Multiplier $(\lambda)$ | Mutation Time = Day 45 | | | | | |  | Mutation Time = Day 50 | | | | | |
| --- | --- | --- | --- | --- | --- | --- | --- | --- | --- | --- | --- | --- | --- |
|  | $H_{i}H_{f}$ | $H_{i}M_{f}$ | $H_{i}L_{f}$ | $M_{i}M_{f}$ | $M_{i}L_{f}$ | $L_{i}L_{f}$ |  | $H_{i}H_{f}$ | $H_{i}M_{f}$ | $H_{i}L_{f}$ | $M_{i}M_{f}$ | $M_{i}L_{f}$ | $L_{i}L_{f}$ |
| 3 | 69.72 | 70.27 | 70.55 | 71.95 | 72.18 | 73.04 |  | 69.27 | 69.79 | 70.06 | 71.85 | 72.06 | 73.12 |
| 2.8 | 71.01 | 71.53 | 71.78 | 73.06 | 73.28 | 74.06 |  | 70.61 | 71.09 | 71.34 | 72.98 | 73.18 | 74.15 |
| 2.6 | 72.30 | 72.78 | 73.02 | 74.17 | 74.37 | 75.08 |  | 71.94 | 72.39 | 72.61 | 74.12 | 74.30 | 75.19 |
| 2.4 | 73.58 | 74.03 | 74.25 | 75.28 | 75.47 | 76.11 |  | 73.26 | 73.68 | 73.88 | 75.25 | 75.42 | 76.23 |
| 2.2 | 74.86 | 75.27 | 75.48 | 76.40 | 76.58 | 77.15 |  | 74.59 | 74.96 | 75.15 | 76.38 | 76.54 | 77.27 |
| 2 | 76.14 | 76.52 | 76.70 | 77.52 | 77.68 | 78.19 |  | 75.90 | 76.25 | 76.42 | 77.52 | 77.66 | 78.31 |
| 1.8 | 77.42 | 77.76 | 77.93 | 78.64 | 78.79 | 79.24 |  | 77.22 | 77.53 | 77.68 | 78.65 | 78.78 | 79.36 |
| 1.6 | 78.70 | 79.00 | 79.16 | 79.77 | 79.90 | 80.29 |  | 78.52 | 78.80 | 78.94 | 79.79 | 79.91 | 80.41 |
| 1.4 | 79.97 | 80.25 | 80.38 | 80.90 | 81.02 | 81.35 |  | 79.83 | 80.08 | 80.20 | 80.93 | 81.03 | 81.47 |
| 1.2 | 81.25 | 81.49 | 81.61 | 82.03 | 82.14 | 82.42 |  | 81.14 | 81.35 | 81.46 | 82.07 | 82.16 | 82.53 |
| 1 | 82.52 | 82.73 | 82.84 | 83.17 | 83.27 | 83.49 |  | 82.44 | 82.62 | 82.71 | 83.21 | 83.29 | 83.59 |

| Capacity Multiplier $(\lambda)$ | Mutation Time = Day 55 | | | | | |  | Mutation Time = Day 60 | | | | | |
| --- | --- | --- | --- | --- | --- | --- | --- | --- | --- | --- | --- | --- | --- |
|  | $H_{i}H_{f}$ | $H_{i}M_{f}$ | $H_{i}L_{f}$ | $M_{i}M_{f}$ | $M_{i}L_{f}$ | $L_{i}L_{f}$ |  | $H_{i}H_{f}$ | $H_{i}M_{f}$ | $H_{i}L_{f}$ | $M_{i}M_{f}$ | $M_{i}L_{f}$ | $L_{i}L_{f}$ |
| 3 | 68.42 | 68.93 | 69.18 | 71.39 | 71.59 | 72.87 |  | 67.25 | 67.74 | 67.99 | 70.64 | 70.83 | 72.34 |
| 2.8 | 69.82 | 70.29 | 70.52 | 72.56 | 72.75 | 73.93 |  | 68.72 | 69.17 | 69.40 | 71.87 | 72.04 | 73.44 |
| 2.6 | 71.21 | 71.64 | 71.86 | 73.74 | 73.90 | 74.99 |  | 70.19 | 70.60 | 70.81 | 73.09 | 73.25 | 74.55 |
| 2.4 | 72.60 | 72.99 | 73.19 | 74.91 | 75.06 | 76.06 |  | 71.65 | 72.02 | 72.21 | 74.31 | 74.46 | 75.65 |
| 2.2 | 73.98 | 74.33 | 74.51 | 76.08 | 76.22 | 77.12 |  | 73.11 | 73.44 | 73.61 | 75.53 | 75.67 | 76.75 |
| 2 | 75.35 | 75.68 | 75.84 | 77.25 | 77.38 | 78.19 |  | 74.56 | 74.86 | 75.01 | 76.75 | 76.87 | 77.86 |
| 1.8 | 76.73 | 77.01 | 77.16 | 78.41 | 78.53 | 79.26 |  | 76.00 | 76.27 | 76.41 | 77.97 | 78.08 | 78.96 |
| 1.6 | 78.09 | 78.35 | 78.48 | 79.58 | 79.69 | 80.33 |  | 77.45 | 77.68 | 77.80 | 79.19 | 79.29 | 80.07 |
| 1.4 | 79.45 | 79.68 | 79.79 | 80.75 | 80.85 | 81.40 |  | 78.89 | 79.09 | 79.19 | 80.41 | 80.50 | 81.17 |
| 1.2 | 80.81 | 81.01 | 81.10 | 81.92 | 82.01 | 82.47 |  | 80.33 | 80.50 | 80.59 | 81.63 | 81.70 | 82.28 |
| 1 | 82.17 | 82.33 | 82.42 | 83.09 | 83.16 | 83.55 |  | 81.76 | 81.91 | 81.98 | 82.85 | 82.91 | 83.39 |

Table S7: Extended model simulation results when the objective is to minimize infection attack rate (IAR) under different vaccine types, capacity multipliers ($\lambda$), and mutation times

| Capacity Multiplier $(\lambda)$ | Mutation Time = Day 5 | | | | | |  | Mutation Time = Day 10 | | | | | |
| --- | --- | --- | --- | --- | --- | --- | --- | --- | --- | --- | --- | --- | --- |
|  | $H_{i}H_{f}$ | $H_{i}M_{f}$ | $H_{i}L_{f}$ | $M_{i}M_{f}$ | $M_{i}L_{f}$ | $L_{i}L_{f}$ |  | $H_{i}H_{f}$ | $H_{i}M_{f}$ | $H_{i}L_{f}$ | $M_{i}M_{f}$ | $M_{i}L_{f}$ | $L_{i}L_{f}$ |
| 3 | 0.8509 | 0.8807 | 0.895 | 0.8814 | 0.8956 | 0.8959 |  | 0.8712 | 0.8959 | 0.9079 | 0.8976 | 0.9095 | 0.9102 |
| 2.8 | 0.8772 | 0.9053 | 0.9187 | 0.9059 | 0.9193 | 0.9196 |  | 0.896 | 0.9194 | 0.9307 | 0.9208 | 0.9321 | 0.9328 |
| 2.6 | 0.9041 | 0.9305 | 0.9431 | 0.931 | 0.9436 | 0.9438 |  | 0.9214 | 0.9434 | 0.9541 | 0.9447 | 0.9553 | 0.9559 |
| 2.4 | 0.9316 | 0.9562 | 0.9679 | 0.9566 | 0.9684 | 0.9686 |  | 0.9474 | 0.968 | 0.978 | 0.9691 | 0.979 | 0.9795 |
| 2.2 | 0.9598 | 0.9825 | 0.9934 | 0.9829 | 0.9937 | 0.9939 |  | 0.974 | 0.9931 | 1.0024 | 0.994 | 1.0033 | 1.0037 |
| 2 | 0.9886 | 1.0094 | 1.0193 | 1.0097 | 1.0196 | 1.0198 |  | 1.0012 | 1.0189 | 1.0274 | 1.0196 | 1.0281 | 1.0285 |
| 1.8 | 1.018 | 1.0368 | 1.0459 | 1.0371 | 1.0461 | 1.0462 |  | 1.0291 | 1.0452 | 1.053 | 1.0458 | 1.0536 | 1.0539 |
| 1.6 | 1.0479 | 1.0648 | 1.073 | 1.065 | 1.0732 | 1.0733 |  | 1.0576 | 1.0721 | 1.0791 | 1.0726 | 1.0796 | 1.0798 |
| 1.4 | 1.0785 | 1.0934 | 1.1006 | 1.0936 | 1.1008 | 1.1009 |  | 1.0867 | 1.0996 | 1.1059 | 1.1 | 1.1063 | 1.1065 |
| 1.2 | 1.1098 | 1.1226 | 1.1289 | 1.1228 | 1.129 | 1.1291 |  | 1.1165 | 1.1278 | 1.1333 | 1.1281 | 1.1335 | 1.1337 |
| 1 | 1.1416 | 1.1524 | 1.1577 | 1.1525 | 1.1578 | 1.1578 |  | 1.147 | 1.1566 | 1.1612 | 1.1568 | 1.1614 | 1.1615 |

| Capacity Multiplier $(\lambda)$ | Mutation Time = Day 15 | | | | | |  | Mutation Time = Day 20 | | | | | |
| --- | --- | --- | --- | --- | --- | --- | --- | --- | --- | --- | --- | --- | --- |
|  | $H_{i}H_{f}$ | $H_{i}M_{f}$ | $H_{i}L_{f}$ | $M_{i}M_{f}$ | $M_{i}L_{f}$ | $L_{i}L_{f}$ |  | $H_{i}H_{f}$ | $H_{i}M_{f}$ | $H_{i}L_{f}$ | $M_{i}M_{f}$ | $M_{i}L_{f}$ | $L_{i}L_{f}$ |
| 3 | 0.8869 | 0.9075 | 0.9176 | 0.9103 | 0.9202 | 0.9216 |  | 0.8985 | 0.9156 | 0.9241 | 0.9198 | 0.9281 | 0.9301 |
| 2.8 | 0.9107 | 0.9302 | 0.9398 | 0.9327 | 0.9421 | 0.9433 |  | 0.9217 | 0.938 | 0.946 | 0.9417 | 0.9495 | 0.9513 |
| 2.6 | 0.935 | 0.9535 | 0.9625 | 0.9556 | 0.9645 | 0.9656 |  | 0.9453 | 0.9608 | 0.9684 | 0.9641 | 0.9715 | 0.9731 |
| 2.4 | 0.9599 | 0.9773 | 0.9857 | 0.9791 | 0.9875 | 0.9884 |  | 0.9696 | 0.9841 | 0.9913 | 0.987 | 0.994 | 0.9953 |
| 2.2 | 0.9855 | 1.0016 | 1.0095 | 1.0032 | 1.011 | 1.0118 |  | 0.9944 | 1.008 | 1.0147 | 1.0104 | 1.017 | 1.0181 |
| 2 | 1.0116 | 1.0265 | 1.0338 | 1.0279 | 1.0351 | 1.0357 |  | 1.0198 | 1.0324 | 1.0386 | 1.0344 | 1.0406 | 1.0415 |
| 1.8 | 1.0383 | 1.052 | 1.0587 | 1.0531 | 1.0598 | 1.0603 |  | 1.0457 | 1.0574 | 1.0631 | 1.059 | 1.0647 | 1.0655 |
| 1.6 | 1.0657 | 1.0781 | 1.0842 | 1.079 | 1.085 | 1.0855 |  | 1.0723 | 1.0829 | 1.0881 | 1.0843 | 1.0894 | 1.0901 |
| 1.4 | 1.0937 | 1.1048 | 1.1103 | 1.1055 | 1.1109 | 1.1113 |  | 1.0995 | 1.1091 | 1.1137 | 1.1101 | 1.1148 | 1.1153 |
| 1.2 | 1.1224 | 1.1322 | 1.137 | 1.1327 | 1.1375 | 1.1377 |  | 1.1274 | 1.1358 | 1.14 | 1.1366 | 1.1407 | 1.1411 |
| 1 | 1.1518 | 1.1602 | 1.1643 | 1.1605 | 1.1646 | 1.1648 |  | 1.156 | 1.1632 | 1.1668 | 1.1638 | 1.1674 | 1.1676 |

| Capacity Multiplier $(\lambda)$ | Mutation Time = Day 25 | | | | | |  | Mutation Time = Day 30 | | | | | |
| --- | --- | --- | --- | --- | --- | --- | --- | --- | --- | --- | --- | --- | --- |
|  | $H_{i}H_{f}$ | $H_{i}M_{f}$ | $H_{i}L_{f}$ | $M_{i}M_{f}$ | $M_{i}L_{f}$ | $L_{i}L_{f}$ |  | $H_{i}H_{f}$ | $H_{i}M_{f}$ | $H_{i}L_{f}$ | $M_{i}M_{f}$ | $M_{i}L_{f}$ | $L_{i}L_{f}$ |
| 3 | 0.9062 | 0.9205 | 0.9277 | 0.9263 | 0.9332 | 0.9359 |  | 0.9103 | 0.9224 | 0.9284 | 0.9299 | 0.9356 | 0.9392 |
| 2.8 | 0.9291 | 0.9428 | 0.9496 | 0.9479 | 0.9545 | 0.9569 |  | 0.9333 | 0.9448 | 0.9506 | 0.9515 | 0.9570 | 0.9602 |
| 2.6 | 0.9525 | 0.9655 | 0.9719 | 0.9700 | 0.9762 | 0.9784 |  | 0.9567 | 0.9677 | 0.9731 | 0.9736 | 0.9788 | 0.9817 |
| 2.4 | 0.9764 | 0.9887 | 0.9947 | 0.9926 | 0.9985 | 1.0004 |  | 0.9806 | 0.9909 | 0.9961 | 0.9961 | 1.0011 | 1.0036 |
| 2.2 | 1.0009 | 1.0123 | 1.0180 | 1.0157 | 1.0213 | 1.0229 |  | 1.0050 | 1.0147 | 1.0195 | 1.0192 | 1.0238 | 1.0260 |
| 2 | 1.0258 | 1.0365 | 1.0418 | 1.0394 | 1.0445 | 1.0459 |  | 1.0298 | 1.0388 | 1.0434 | 1.0427 | 1.0470 | 1.0489 |
| 1.8 | 1.0513 | 1.0612 | 1.0661 | 1.0636 | 1.0684 | 1.0695 |  | 1.0552 | 1.0635 | 1.0677 | 1.0667 | 1.0708 | 1.0723 |
| 1.6 | 1.0774 | 1.0864 | 1.0909 | 1.0884 | 1.0928 | 1.0937 |  | 1.0810 | 1.0887 | 1.0925 | 1.0913 | 1.0950 | 1.0963 |
| 1.4 | 1.1041 | 1.1122 | 1.1163 | 1.1138 | 1.1178 | 1.1185 |  | 1.1074 | 1.1144 | 1.1178 | 1.1165 | 1.1199 | 1.1209 |
| 1.2 | 1.1314 | 1.1386 | 1.1422 | 1.1398 | 1.1434 | 1.1439 |  | 1.1344 | 1.1406 | 1.1437 | 1.1422 | 1.1452 | 1.1460 |
| 1 | 1.1594 | 1.1656 | 1.1687 | 1.1665 | 1.1696 | 1.1700 |  | 1.1620 | 1.1673 | 1.1700 | 1.1686 | 1.1712 | 1.1718 |

| Capacity Multiplier $(\lambda)$ | Mutation Time = Day 35 | | | | | |  | Mutation Time = Day 40 | | | | | |
| --- | --- | --- | --- | --- | --- | --- | --- | --- | --- | --- | --- | --- | --- |
|  | $H_{i}H_{f}$ | $H_{i}M_{f}$ | $H_{i}L_{f}$ | $M_{i}M_{f}$ | $M_{i}L_{f}$ | $L_{i}L_{f}$ |  | $H_{i}H_{f}$ | $H_{i}M_{f}$ | $H_{i}L_{f}$ | $M_{i}M_{f}$ | $M_{i}L_{f}$ | $L_{i}L_{f}$ |
| 3 | 0.9111 | 0.9213 | 0.9265 | 0.9308 | 0.9356 | 0.9401 |  | 0.9087 | 0.9175 | 0.9220 | 0.9291 | 0.9332 | 0.9387 |
| 2.8 | 0.9344 | 0.9442 | 0.9491 | 0.9527 | 0.9573 | 0.9613 |  | 0.9326 | 0.9409 | 0.9452 | 0.9514 | 0.9553 | 0.9603 |
| 2.6 | 0.9581 | 0.9674 | 0.9721 | 0.9749 | 0.9793 | 0.9829 |  | 0.9568 | 0.9647 | 0.9688 | 0.9741 | 0.9777 | 0.9822 |
| 2.4 | 0.9823 | 0.9910 | 0.9954 | 0.9976 | 1.0018 | 1.0050 |  | 0.9814 | 0.9888 | 0.9926 | 0.9971 | 1.0006 | 1.0046 |
| 2.2 | 1.0068 | 1.0150 | 1.0191 | 1.0208 | 1.0247 | 1.0274 |  | 1.0063 | 1.0133 | 1.0169 | 1.0206 | 1.0238 | 1.0273 |
| 2 | 1.0318 | 1.0394 | 1.0433 | 1.0444 | 1.0480 | 1.0504 |  | 1.0316 | 1.0382 | 1.0415 | 1.0444 | 1.0475 | 1.0505 |
| 1.8 | 1.0572 | 1.0643 | 1.0678 | 1.0684 | 1.0718 | 1.0739 |  | 1.0573 | 1.0634 | 1.0664 | 1.0687 | 1.0716 | 1.0741 |
| 1.6 | 1.0830 | 1.0895 | 1.0928 | 1.0930 | 1.0962 | 1.0978 |  | 1.0835 | 1.0890 | 1.0918 | 1.0934 | 1.0961 | 1.0982 |
| 1.4 | 1.1094 | 1.1153 | 1.1183 | 1.1181 | 1.1210 | 1.1223 |  | 1.1100 | 1.1150 | 1.1175 | 1.1187 | 1.1211 | 1.1228 |
| 1.2 | 1.1363 | 1.1415 | 1.1442 | 1.1438 | 1.1463 | 1.1474 |  | 1.1370 | 1.1415 | 1.1437 | 1.1444 | 1.1465 | 1.1479 |
| 1 | 1.1637 | 1.1683 | 1.1706 | 1.1700 | 1.1722 | 1.1730 |  | 1.1644 | 1.1683 | 1.1703 | 1.1706 | 1.1725 | 1.1736 |

| Capacity Multiplier $(\lambda)$ | Mutation Time = Day 45 | | | | | |  | Mutation Time = Day 50 | | | | | |
| --- | --- | --- | --- | --- | --- | --- | --- | --- | --- | --- | --- | --- | --- |
|  | $H_{i}H_{f}$ | $H_{i}M_{f}$ | $H_{i}L_{f}$ | $M_{i}M_{f}$ | $M_{i}L_{f}$ | $L_{i}L_{f}$ |  | $H_{i}H_{f}$ | $H_{i}M_{f}$ | $H_{i}L_{f}$ | $M_{i}M_{f}$ | $M_{i}L_{f}$ | $L_{i}L_{f}$ |
| 3 | 0.9036 | 0.9112 | 0.9151 | 0.9251 | 0.9286 | 0.9352 |  | 0.8961 | 0.9026 | 0.9061 | 0.9191 | 0.9220 | 0.9298 |
| 2.8 | 0.9282 | 0.9353 | 0.9391 | 0.9480 | 0.9512 | 0.9573 |  | 0.9214 | 0.9276 | 0.9309 | 0.9425 | 0.9453 | 0.9525 |
| 2.6 | 0.9530 | 0.9598 | 0.9633 | 0.9711 | 0.9742 | 0.9797 |  | 0.9470 | 0.9529 | 0.9560 | 0.9664 | 0.9690 | 0.9755 |
| 2.4 | 0.9782 | 0.9846 | 0.9879 | 0.9947 | 0.9976 | 1.0025 |  | 0.9729 | 0.9785 | 0.9813 | 0.9905 | 0.9930 | 0.9988 |
| 2.2 | 1.0037 | 1.0097 | 1.0128 | 1.0186 | 1.0213 | 1.0256 |  | 0.9991 | 1.0043 | 1.0070 | 1.0150 | 1.0173 | 1.0225 |
| 2 | 1.0296 | 1.0351 | 1.0380 | 1.0428 | 1.0454 | 1.0492 |  | 1.0257 | 1.0304 | 1.0329 | 1.0398 | 1.0420 | 1.0465 |
| 1.8 | 1.0557 | 1.0609 | 1.0635 | 1.0675 | 1.0699 | 1.0731 |  | 1.0525 | 1.0569 | 1.0591 | 1.0650 | 1.0670 | 1.0709 |
| 1.6 | 1.0823 | 1.0870 | 1.0894 | 1.0926 | 1.0948 | 1.0975 |  | 1.0796 | 1.0836 | 1.0857 | 1.0905 | 1.0924 | 1.0957 |
| 1.4 | 1.1092 | 1.1134 | 1.1156 | 1.1181 | 1.1201 | 1.1224 |  | 1.1070 | 1.1106 | 1.1125 | 1.1164 | 1.1181 | 1.1209 |
| 1.2 | 1.1365 | 1.1403 | 1.1422 | 1.1440 | 1.1458 | 1.1476 |  | 1.1348 | 1.1380 | 1.1396 | 1.1427 | 1.1442 | 1.1465 |
| 1 | 1.1641 | 1.1675 | 1.1692 | 1.1704 | 1.1720 | 1.1734 |  | 1.1629 | 1.1657 | 1.1671 | 1.1694 | 1.1708 | 1.1726 |

| Capacity Multiplier $(\lambda)$ | Mutation Time = Day 55 | | | | | |  | Mutation Time = Day 60 | | | | | |
| --- | --- | --- | --- | --- | --- | --- | --- | --- | --- | --- | --- | --- | --- |
|  | $H_{i}H_{f}$ | $H_{i}M_{f}$ | $H_{i}L_{f}$ | $M_{i}M_{f}$ | $M_{i}L_{f}$ | $L_{i}L_{f}$ |  | $H_{i}H_{f}$ | $H_{i}M_{f}$ | $H_{i}L_{f}$ | $M_{i}M_{f}$ | $M_{i}L_{f}$ | $L_{i}L_{f}$ |
| 3 | 0.8866 | 0.8924 | 0.8954 | 0.9114 | 0.9138 | 0.9229 |  | 0.8760 | 0.8810 | 0.8837 | 0.9026 | 0.9047 | 0.9151 |
| 2.8 | 0.9128 | 0.9182 | 0.9211 | 0.9356 | 0.9379 | 0.9462 |  | 0.9030 | 0.9077 | 0.9102 | 0.9275 | 0.9295 | 0.9390 |
| 2.6 | 0.9392 | 0.9443 | 0.9470 | 0.9601 | 0.9623 | 0.9699 |  | 0.9302 | 0.9346 | 0.9370 | 0.9527 | 0.9546 | 0.9633 |
| 2.4 | 0.9659 | 0.9707 | 0.9732 | 0.9849 | 0.9870 | 0.9938 |  | 0.9578 | 0.9619 | 0.9641 | 0.9782 | 0.9800 | 0.9879 |
| 2.2 | 0.9929 | 0.9974 | 0.9997 | 1.0100 | 1.0120 | 1.0181 |  | 0.9856 | 0.9894 | 0.9914 | 1.0041 | 1.0057 | 1.0128 |
| 2 | 1.0202 | 1.0243 | 1.0265 | 1.0354 | 1.0373 | 1.0427 |  | 1.0136 | 1.0172 | 1.0190 | 1.0302 | 1.0317 | 1.0380 |
| 1.8 | 1.0477 | 1.0515 | 1.0535 | 1.0612 | 1.0629 | 1.0676 |  | 1.0420 | 1.0452 | 1.0469 | 1.0566 | 1.0580 | 1.0636 |
| 1.6 | 1.0756 | 1.0790 | 1.0808 | 1.0873 | 1.0889 | 1.0929 |  | 1.0706 | 1.0735 | 1.0750 | 1.0833 | 1.0846 | 1.0895 |
| 1.4 | 1.1036 | 1.1067 | 1.1083 | 1.1138 | 1.1152 | 1.1186 |  | 1.0994 | 1.1020 | 1.1034 | 1.1104 | 1.1116 | 1.1157 |
| 1.2 | 1.1320 | 1.1348 | 1.1362 | 1.1406 | 1.1418 | 1.1447 |  | 1.1285 | 1.1308 | 1.1320 | 1.1378 | 1.1388 | 1.1422 |
| 1 | 1.1607 | 1.1631 | 1.1643 | 1.1677 | 1.1688 | 1.1711 |  | 1.1579 | 1.1598 | 1.1609 | 1.1655 | 1.1664 | 1.1692 |

Table S8: Extended model simulation results when the objective is to minimize the mortality rate under different vaccine types, capacity multipliers ($\lambda$), and mutation times

Figure S3 shows the daily new infection (blue) and the individuals who are vaccinated and infected ($I_{A}^{V}$ – red, $I_{S}^{V}$ – green) for all vaccine types when the capacity multiplier ($\lambda$) is 3.0 and the mutation time is on day 20, 40, and 60. As the mutation time occurs at a time closer to and before the daily infection peak, a large number of susceptible individuals (i.e., never vaccinated + vaccinated before the mutation time) gets exposed to a quickly growing number of infected individuals. Hence, a higher IAR is estimated compared to when the mutation time occurs earlier.


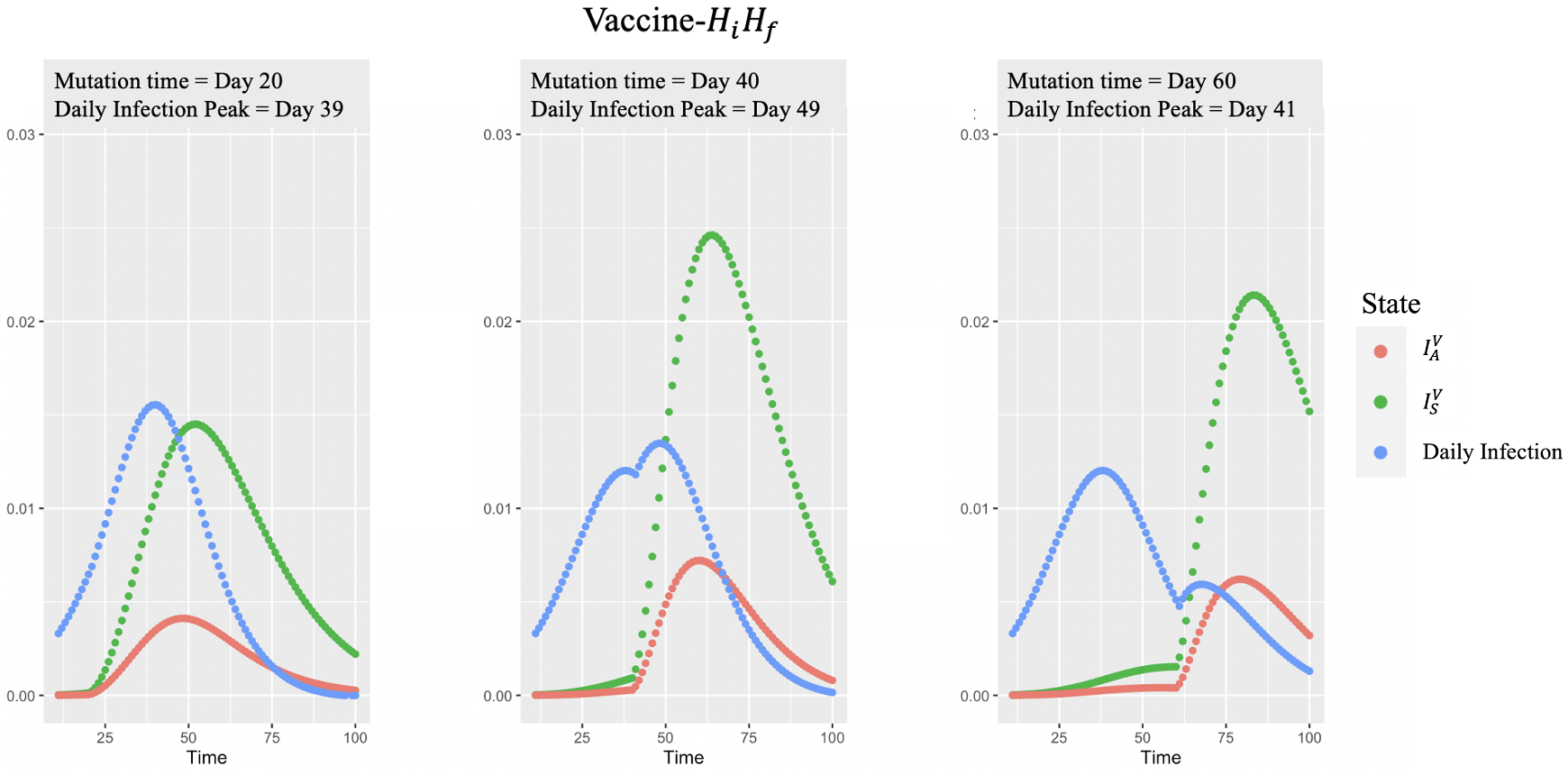


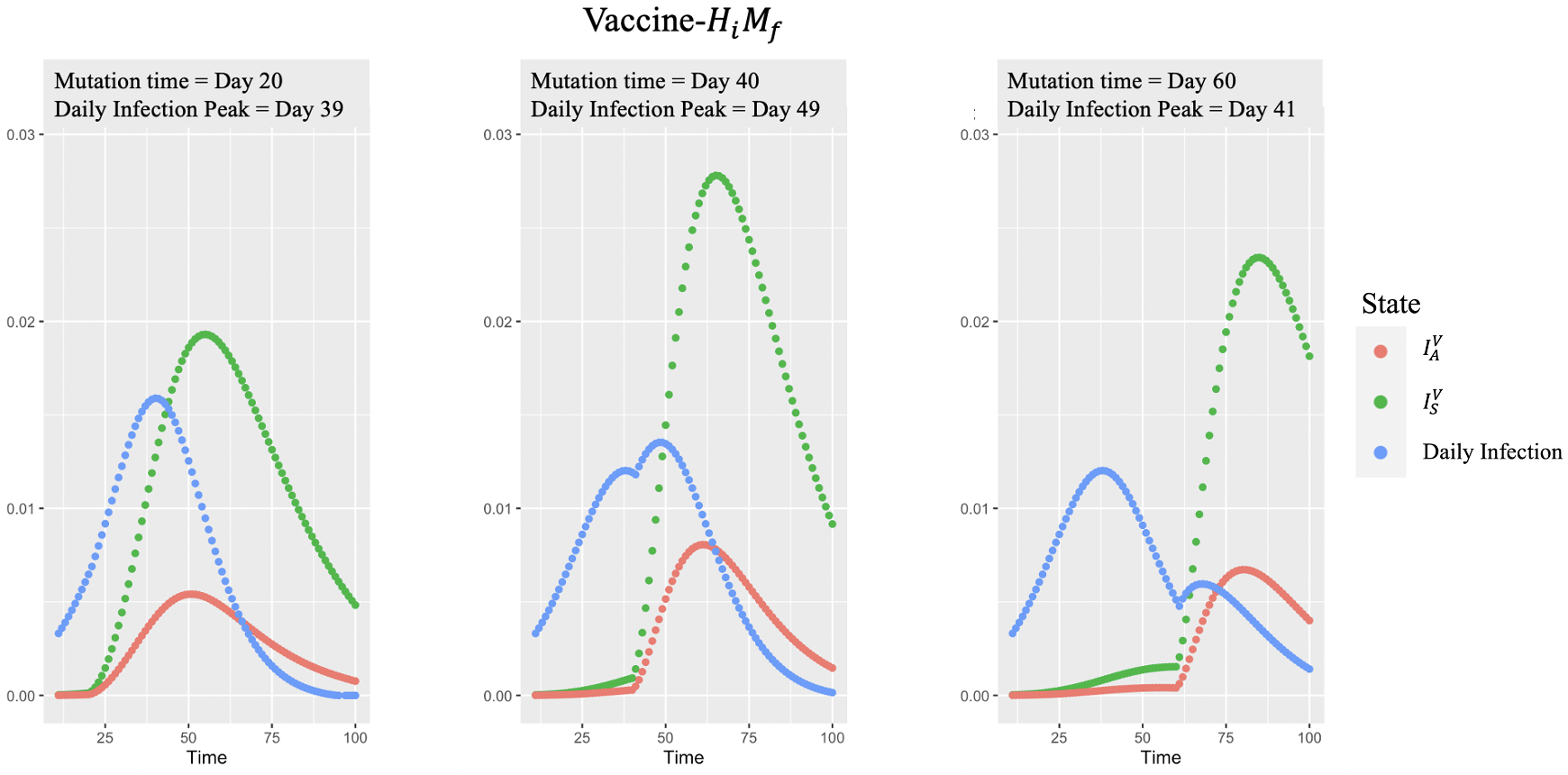


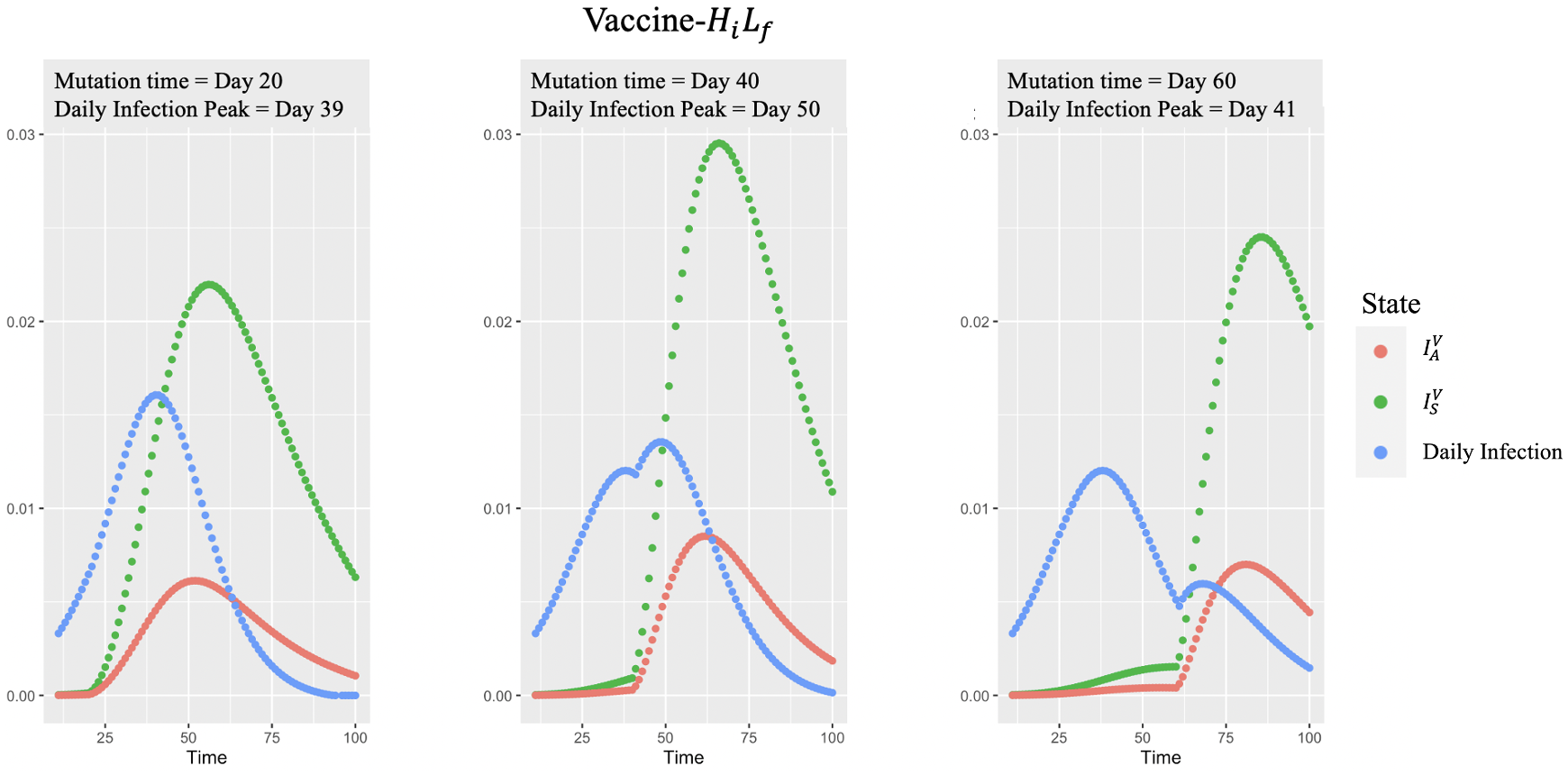


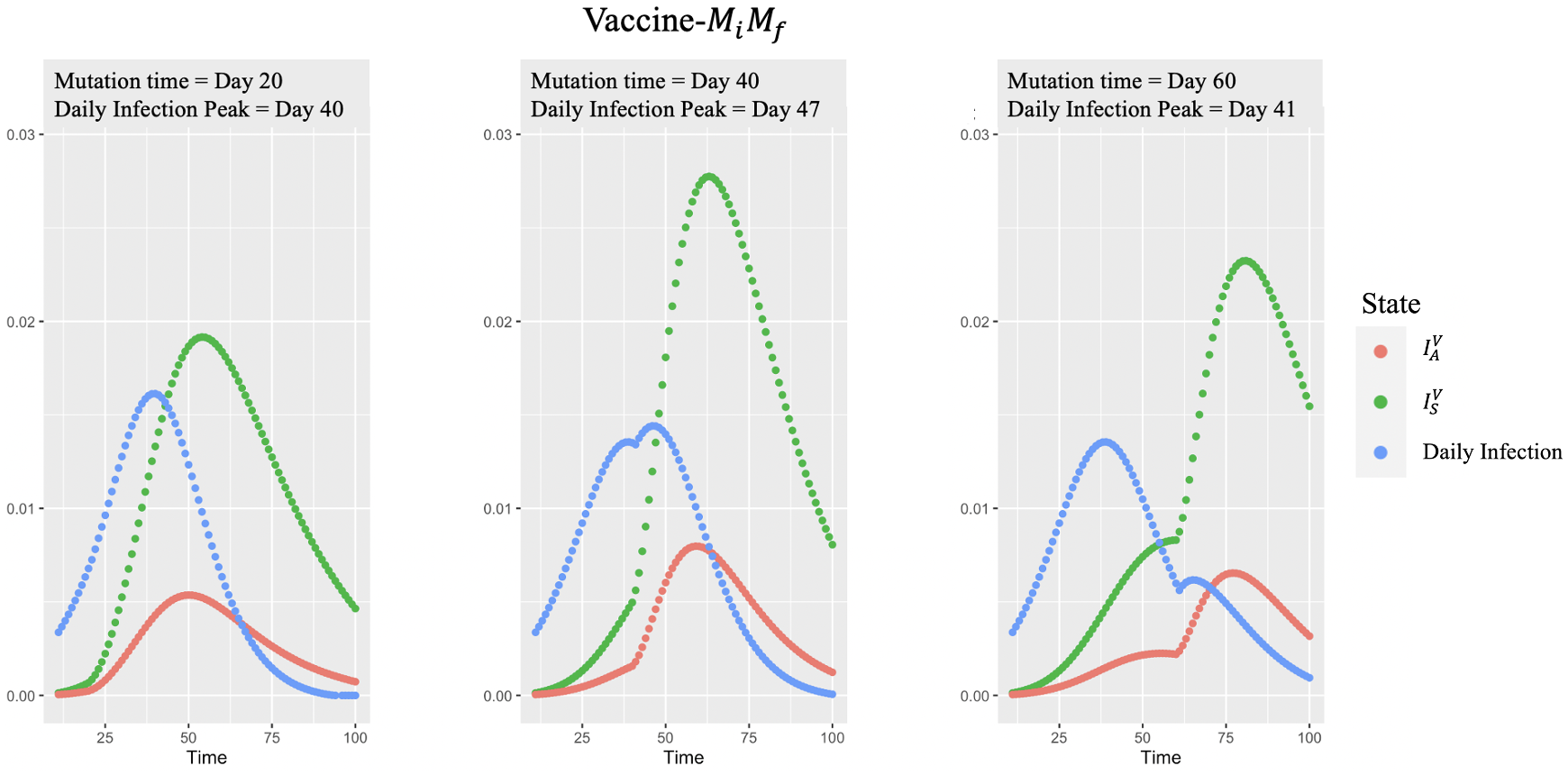


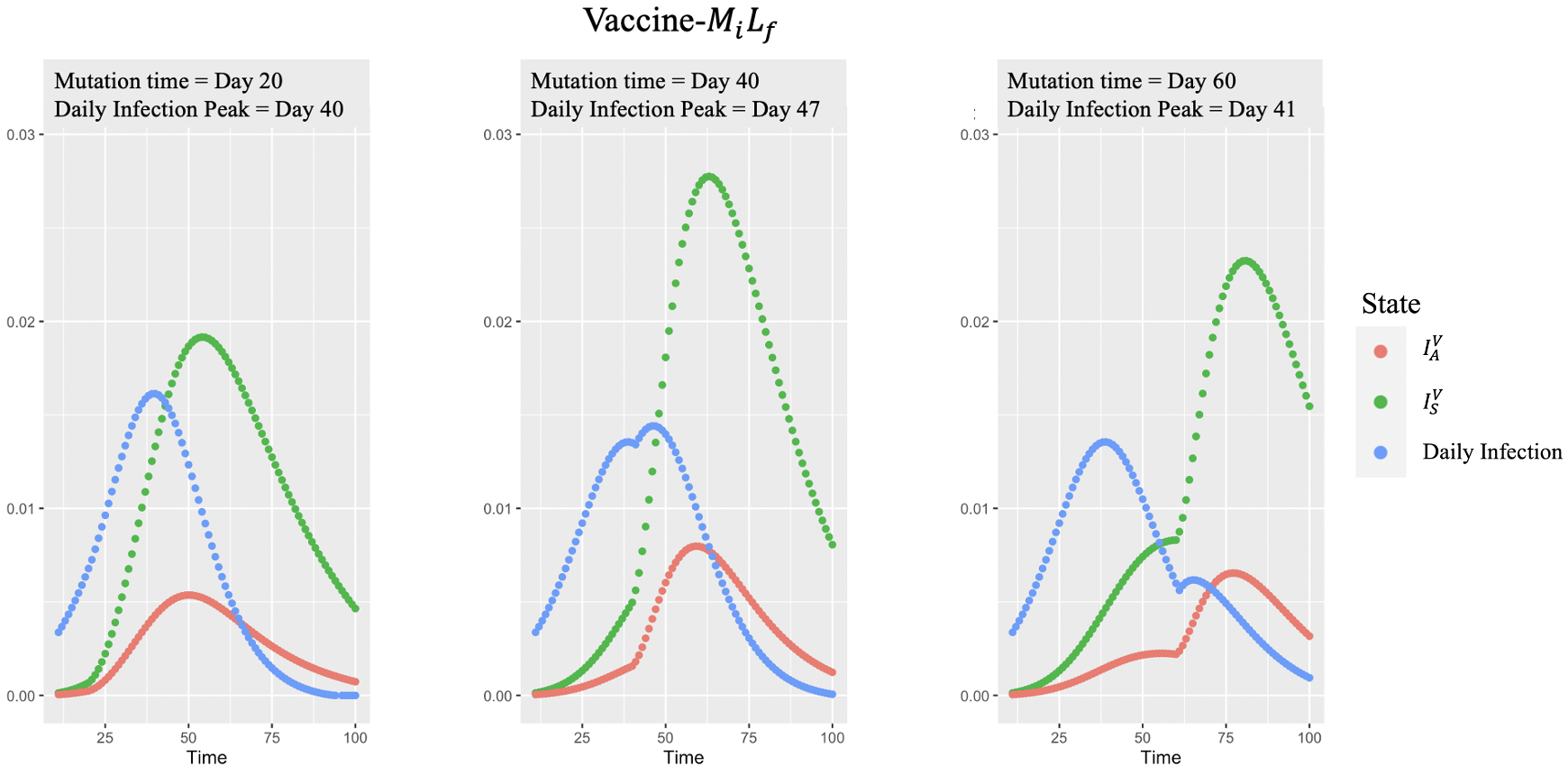


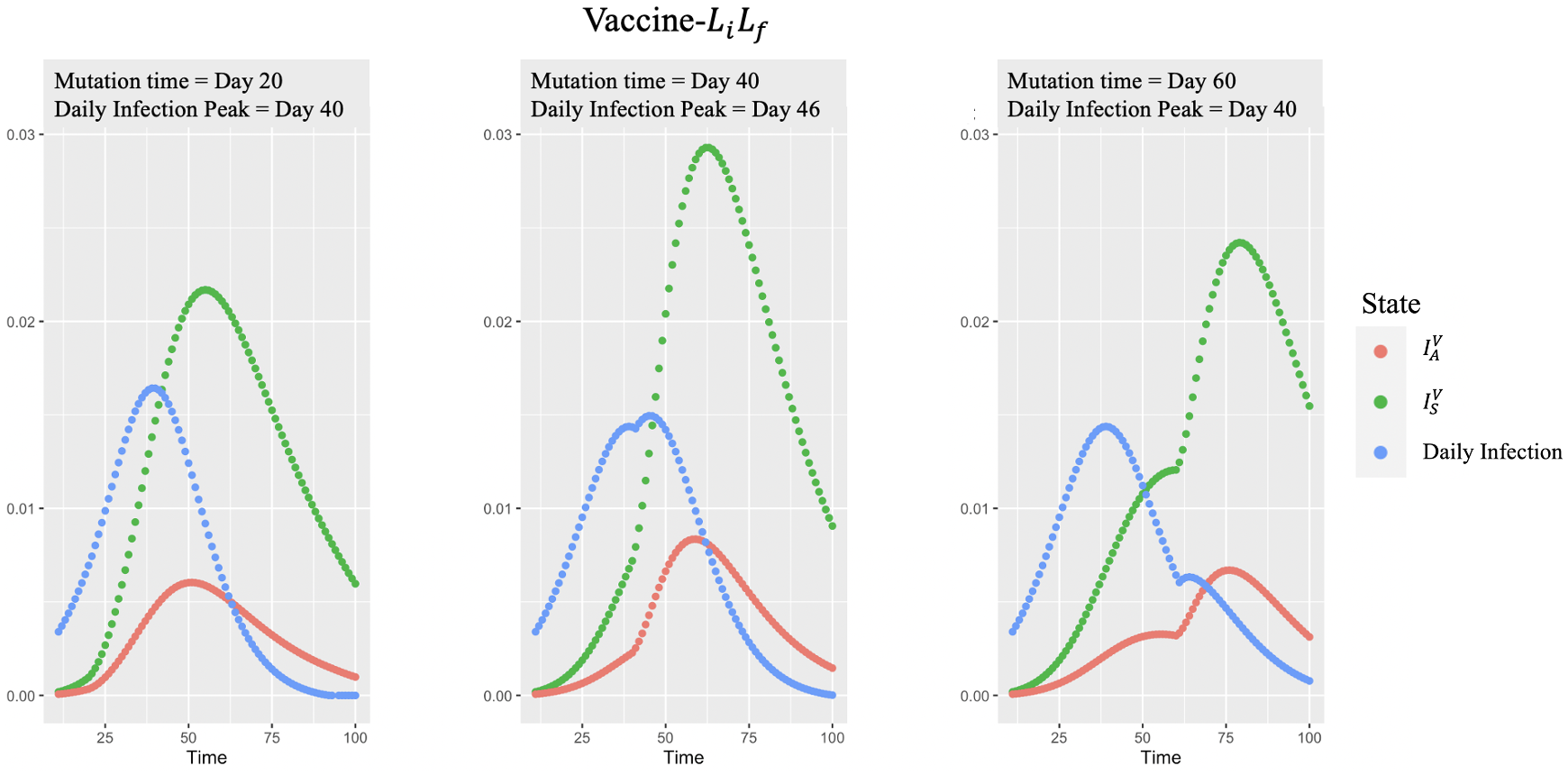


Figure S3: Daily new infections and vaccinated but infected populations from day 15 to day 100 with all vaccine types when the capacity multiplier ($\lambda$) is 3 and the mutation time is on day 20, 40, and 60 in the extended model

1 Food and Drug Administration. *Comirnaty and Pfizer-BioNTech COVID-19 Vaccine*, <<https://www.fda.gov/emergency-preparedness-and-response/coronavirus-disease-2019-covid-19/comirnaty-and-pfizer-biontech-covid-19-vaccine>> (2022).

2 Xu, S. *et al.* COVID-19 Vaccination and Non-COVID-19 Mortality Risk - Seven Integrated Health Care Organizations, United States, December 14, 2020-July 31, 2021. *MMWR Morb Mortal Wkly Rep* **70**, 1520-1524, doi:10.15585/mmwr.mm7043e2 (2021).

3 Johnson, A. G. COVID-19 incidence and death rates among unvaccinated and fully vaccinated adults with and without booster doses during periods of Delta and Omicron variant emergence—25 US Jurisdictions, April 4–December 25, 2021. *MMWR Morb Mortal Wkly Rep* **71** (2022).

4 Centers for Disease Control and Prevention. *Quarantine and Isolation*, <<https://www.cdc.gov/coronavirus/2019-ncov/your-health/quarantine-isolation.html>> (2022).

1. Since the mutation time, $v_{i}=0$ [↑](#footnote-ref-1)
2. Before the mutation time, $v_{f}=0$ [↑](#footnote-ref-2)
3. $\delta=0$ before the mutation time and $\delta=1/7$ since the mutation time [↑](#footnote-ref-3)
